# Intermediate Filament Protein BFSP1 Maintains Oocyte Asymmetric Division by Modulating Spindle Length

**DOI:** 10.1002/advs.202504066

**Published:** 2025-05-11

**Authors:** Yu Li, Hanwen Zhang, Wenjun Zeng, Yilong Miao, Shaochen Sun, Yu Zhang, Bo Xiong

**Affiliations:** ^1^ College of Animal Science and Technology Nanjing Agricultural University Nanjing 210095 China; ^2^ College of Animal Sciences Zhejiang University Hangzhou 310058 China

**Keywords:** asymmetric division, BFSP1, intermediate filament protein, oocyte meiosis, spindle length

## Abstract

The cytoskeleton is composed of microtubules, microfilaments, and intermediate filaments in cells. While the functions of microtubules and microfilaments have been well elucidated, the roles of intermediate filaments and associated proteins remain largely unknown, especially in meiosis. BFSP1 is an intermediate filament protein mainly expressed in the eye lens to play important roles in the development of congenital cataract. Here, we document that BFSP1 functions as a spindle regulator to drive the oocyte asymmetric division. Specifically, we found that BFSP1 distributed on the spindle apparatus during oocyte meiotic maturation. Depletion of BFSP1 resulted in symmetric division of oocytes, accompanied by the formation of elongated spindles at metaphase I and anaphase/telophase I stages. In addition, immunoprecipitation combined with mass spectrometry analysis identified MAP1B, a microtubule‐associated protein, as an interacting partner of BFSP1. Depletion or mutation of MAP1B phenocopied the meiotic defects observed in BFSP1‐depleted oocytes, and expression of exogenous MAP1B‐EGFP in BFSP1‐depleted oocytes recovered the spindle length and asymmetric division. We further determined that BFSP1 recruited molecular chaperone HSP90α on the spindle to stabilize MAP1B, thereby controlling the spindle length. To sum up, our findings reveal a unique meiotic role for BFSP1 in the regulation of spindle dynamics and oocyte asymmetric division.

## Graphical abstract

Different from mitosis, the female meiosis undergoes asymmetric division that produces haploid oocytes and polar body, which is essential for retaining maternal components to support subsequent fertilization and embryo development. However, the underlying mechanisms are still largely unknown. This work documents that an intermediate filament orphan protein BFSP1, predominantly expressed in lens cells, drives oocyte asymmetric division by modulating spindle length.

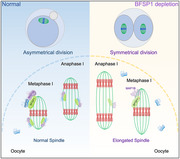

## 1 Introduction

The cytoskeleton of eukaryotic cells is composed of a complex network of fibers, primarily made up of three protein families that assemble into three types of filaments: microtubules, microfilaments, and intermediate filaments. The interactions between cytoskeletal proteins and numerous cytoskeletal binding proteins are the molecular basis for the unity of cell structure and function.^[^ ^1^ ^]^ However, compared to microtubules and microfilaments, much less is known about the third component of the cytoskeleton, intermediate filaments. Intermediate filaments are composed of a variety of distinct protein subunits that form a coiled‐coil dimer, which then assembles into a larger rope‐like structure with 8–12 nm in diameter.^[^ ^2^ ^]^ The composition and properties of intermediate filaments vary depending on the specific protein family, which in turn is regulated by cell type and tissue differentiation.^[^ ^3^ ^]^ The major classes of intermediate filament proteins include keratin (primarily expressed in epithelial cells), vimentin (typically found in mesenchymal cells), desmin (primarily present in muscle cells), neurofilaments (present in neurons), and lamins (main component of the nuclear envelope).^[^ ^4^ ^]^ Intermediate filaments assemble into an extensive cytoskeletal network that appears to connect the cell surface to the nucleus, providing essential mechanical properties to the cell.^[^ ^5^, ^6^, ^7^, ^8^ ^]^ However, intermediate filaments also exert functions beyond mechanical support. Previous studies have shown that the distribution of intermediate filaments provides a large surface area that can serve as a scaffold for the binding of various regulatory and signaling molecules.^[^ ^9^, ^10^, ^11^, ^12^, ^13^ ^]^ In addition, the intermediate filament system has been reported to associate with membrane organelles such as mitochondria, Golgi apparatus, and vesicles, as well as other cytoskeletal components and their associated motor proteins, playing a crucial role in the internal organization and positioning of organelles and other cytoplasmic components.^[^ ^14^, ^15^, ^16^, ^17^ ^]^

Beaded filament structural protein 1 (BFSP1) is a unique protein within the intermediate filament family, classified as an “orphan” gene because of its specific sequence features.^[^ ^18^ ^]^ At present, studies on BFSP1 mainly focus on its role in lens fiber cells of the eye. Similarly, BFSP2 is another intermediate filament orphan protein and is also present in lens fiber cells.^[^ ^19^ ^]^ BFSP1 and BFSP2 proteins assemble into distinctive periodic beaded filament structures which are specifically distributed in differentiated lens fiber cells.^[^ ^20^, ^21^, ^22^, ^23^, ^24^, ^25^ ^]^ Point mutations in BFSP1 can lead to congenital cataracts inherited in an autosomal dominant manner in humans, suggesting that its normal expression is crucial for maintaining the transparency of the lens.^[^ ^26^, ^27^ ^]^ In addition, genetic deletion of BFSP2 in mice results in the impaired protein stability of BFSP1 in lens fiber cells, which in turn disrupts the beaded filament structure in the lens.^[^ ^28^, ^29^ ^]^ However, the expression and function of BFSP1 other than lens fiber cells have not been extensively investigated.

Different from mitosis, the female meiosis undergoes asymmetric division that produces totipotent haploid oocytes and non‐DNA‐replicating polar body, which is essential for retaining maternal components to support subsequent fertilization and embryo development.^[^ ^30^, ^31^, ^32^ ^]^ Extensive cytoplasmic skeletal reorganization occurs during oocyte maturation and fertilization to drive the meiotic events and progression, including meiotic spindle formation and migration, chromosome segregation, polar body extrusion, and pronuclear formation and migration.^[^ ^33^ ^]^ Whether the intermediate filament proteins are involved in these events is largely elusive.

In the present study, we found that BFSP1 distributed on the spindle fibers and interacted with MAP1B to control the spindle length, and thus driving the asymmetric division of oocytes. Moreover, we demonstrated that BFSP1 is required for recruitment of molecular chaperon HSP90α to the spindle for stabilizing MAP1B.

## 2 Results

### 2.1 Expression and Subcellular Localization of BFSP1 During Mouse Oocyte Meiosis

**Figure 1:**
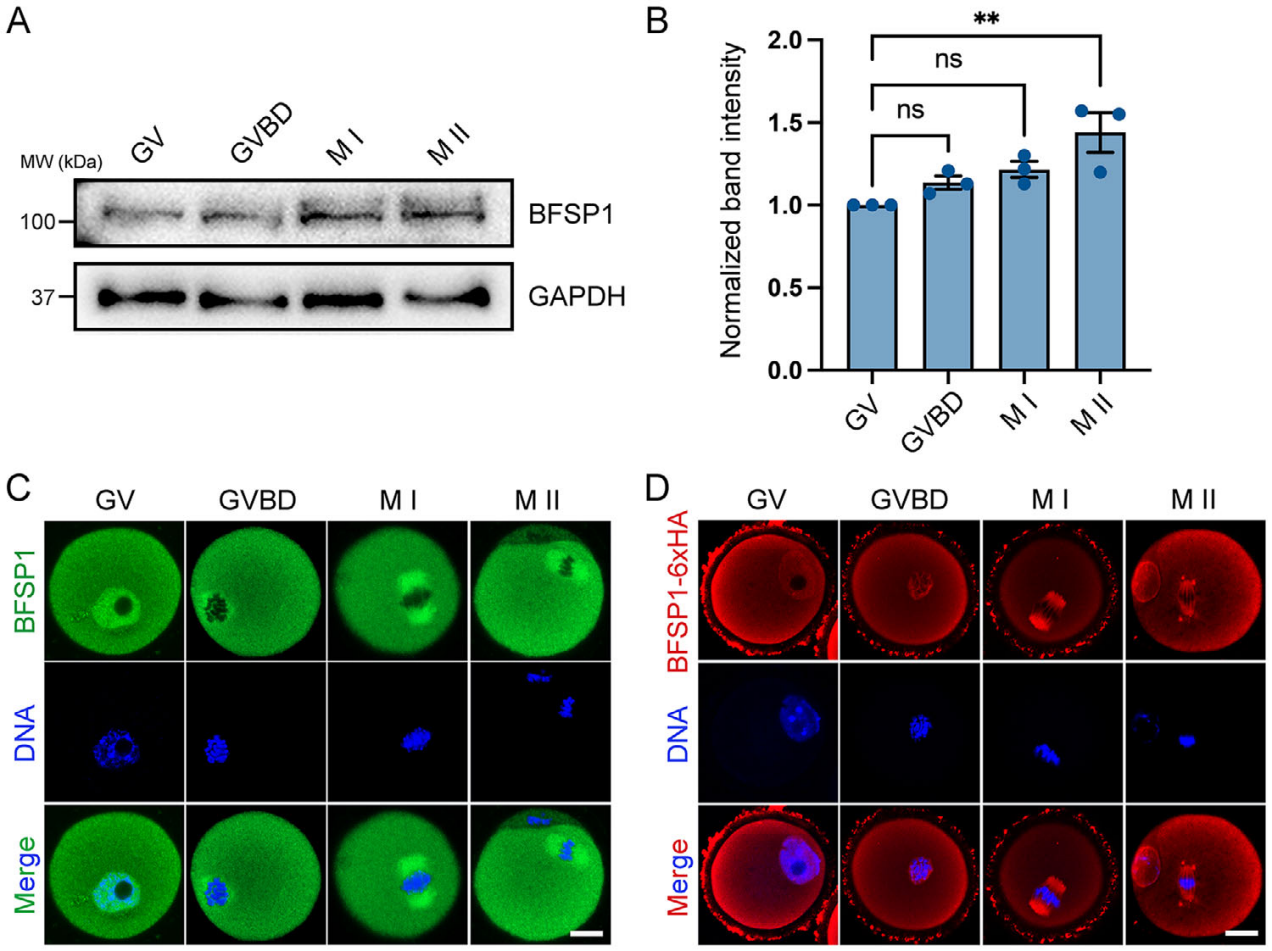
Protein expression and subcellular localization of BFSP1 during mouse oocyte meiosis. A) Protein levels of BFSP1 in oocytes at different developmental stages corresponding to GV (germinal vesicle), GVBD (germinal vesicle breakdown), M I (metaphase I), and M II (metaphase II) were examined by immunoblotting analysis. B) The band intensity of BFSP1 in the blots was normalized with that of GAPDH. C) Representative images of BFSP1 localization during oocyte maturation. Scale bar, 20 µm. D) Representative images of BFSP1‐6×HA localization during oocyte maturation. Scale bar, 20 µm. Data in (B) were expressed as mean ± SEM of at least three independent experiments. ^**^ *P* < 0.01; ns, no significance.

To explore the potential role of BFSP1 during oocyte meiotic progression, we first determined whether BFSP1 is expressed in oocytes at different maturational stages by immunoblotting analysis. The result showed that BFSP1 protein was constantly observed in germinal vesicle (GV), germinal vesicle breakdown (GVBD), metaphase I (M I), and metaphase II (M II) oocytes, and slightly increased after GVBD (**Figure** **1**A,B). We next examined the subcellular localization of endogenous BFSP1 in mouse oocytes by antibody staining. The fluorescence imaging data manifested that BFSP1 was localized in both the nucleus and cytoplasm of GV oocytes. Notably, after GVBD, in addition to the cytoplasmic localization, BFSP1 was predominantly accumulated to the spindle region at both M I and M II stages (Figure 1C). To further confirm this localization pattern, we microinjected exogenous BFSP1‐6×Myc and BFSP1‐6×HA mRNA into oocytes, and then observed their expression and distribution with the tag antibodies. As expected, both BFSP1‐6×Myc and BFSP1‐6×HA exhibited the similar localization pattern with the endogenous BFSP1 (Figure 1D; Figure S1, Supporting Information), suggesting that BFSP1 may play an important role in meiotic spindle dynamics in oocytes.

### 2.2 Depletion of BFSP1 Leads to Oocyte Meiotic Maturation Failure and Symmetric Division

**Figure 2:**
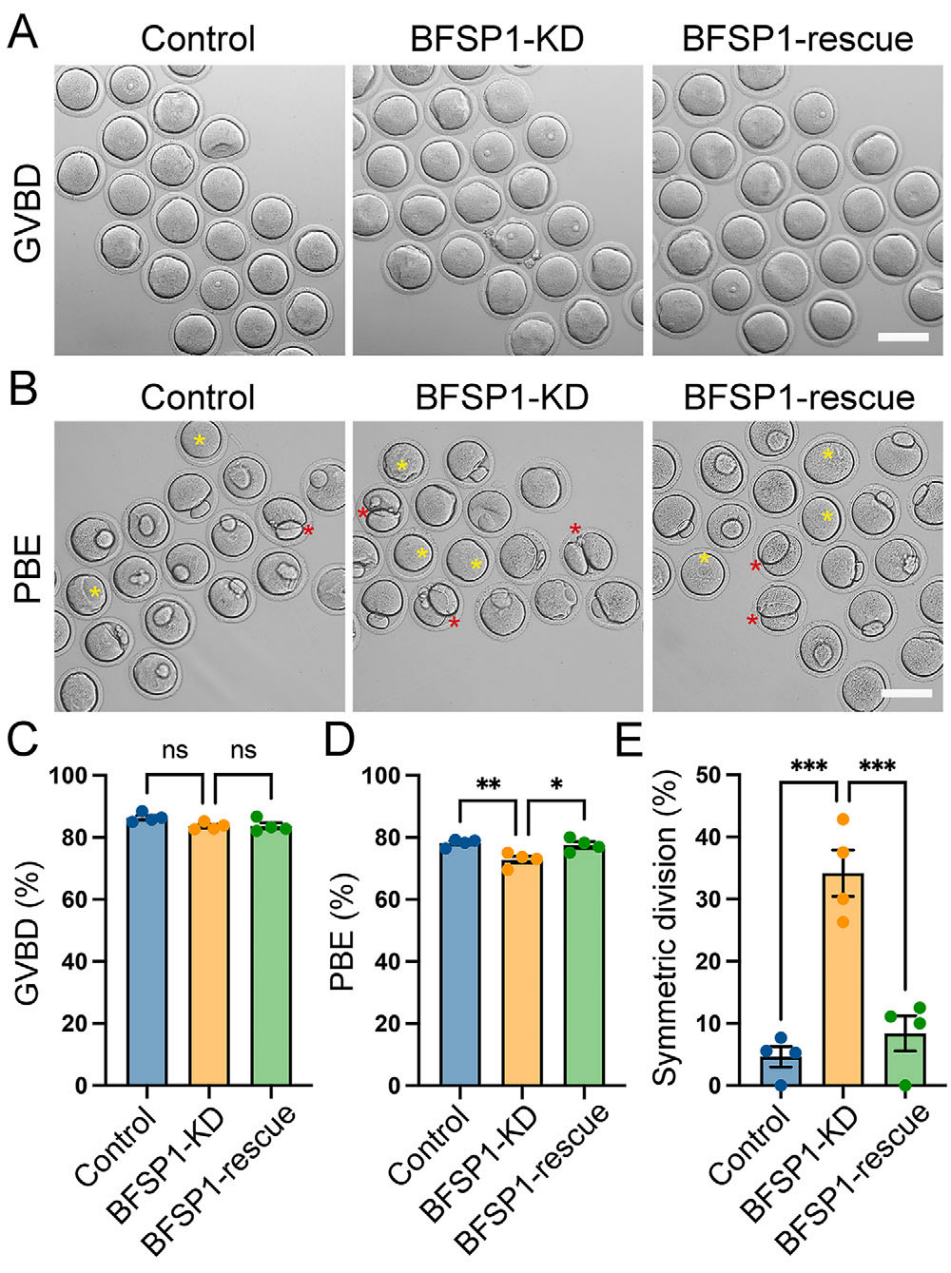
BFSP1 depletion leads to the defective oocyte meiotic maturation. A) Representative images of oocytes at GVBD stage in control, BFSP1‐KD (knockdown), and BFSP1‐rescue groups. Scale bar, 80 µm. B) Representative images of oocytes at M II stage in control, BFSP1‐KD, and BFSP1‐rescue groups. Yellow asterisks indicate oocytes that failed to extrude the first polar body, and red asterisks indicate oocytes with symmetric division. Scale bar, 80 µm. C) The GVBD rate was quantified in control (*n* = 96), BFSP1‐KD (*n* = 110), and BFSP1‐rescue (*n* = 110) oocytes. D) The PBE (polar body extrusion) rate was quantified in control (*n* = 96), BFSP1‐KD (*n* = 110), and BFSP1‐rescue (*n* = 110) oocytes. E) The rate of symmetric division was quantified in control (*n* = 96), BFSP1‐KD (*n* = 110), and BFSP1‐rescue (*n* = 110) oocytes. Data in (C), (D), and (E) were expressed as mean ± SEM of at least three independent experiments. ^*^ *P* < 0.05; ^**^ *P* < 0.01; ^***^ *P* < 0.001; ns, no significance.

We then performed loss‐of‐function experiments by microinjecting BFSP1‐targeting siRNAs into GV oocytes to investigate BFSP1 function during oocyte meiosis. Quantitative real‐time polymerase chain reaction (qRT‐PCR) and immunoblotting analyses revealed that siRNA‐mediated knockdown of BFSP1 resulted in ≈70% downregulation of the target transcript and protein in oocytes (Figure S2A–C, Supporting Information). Immunostaining data also confirmed the reduction of BFSP1 signals in BFSP1‐depleted oocytes (Figure S2D,E, Supporting Information). In the meantime, we found that depletion of BFSP1 had no effect on the protein levels of BFSP2 (Figure S2F,G, Supporting Information). Following knockdown, the meiotic progression of both control and BFSP1‐depleted oocytes were monitored. We found that depletion of BFSP1 did not affect GVBD (**Figure** **2**A,C), but decreased the rate of first polar body extrusion (PBE) (Figure 2B,D), as well as weakened the fertilization ability of oocytes (Figure S3, Supporting Information). In addition, we noticed that a higher proportion of large polar bodies (the polar body width is more than 50% of the oocyte diameter) were present in BFSP1‐depleted oocytes compared to the controls (Figure 2B,E). To exclude the possibility that these meiotic defects were caused by the off‐target effects of siRNA, we also introduced the exogenous BFSP1‐6×HA into BFSP1‐depleted oocytes to restore the BFSP1 protein level and function. As shown in Figure S2H (Supporting Information), expression of BFSP1‐6×HA elevated the protein level of BFSP1 in BFSP1‐depleted oocytes (Figure S2H,I, Supporting Information). Meanwhile, expression of BFSP1‐6×HA significantly increased the rate of PBE and reduced the occurrence of large polar bodies in oocytes depleted of BFSP1 (Figure 2B–E), indicating that these defects indeed resulted from BFSP1 loss‐of‐function. Collectively, our observations demonstrate that BFSP1 plays a critical role in the oocyte meiotic maturation and asymmetric division.

### 2.3 Depletion of BFSP1 Results in an Elongated Spindle in Oocytes

**Figure 3:**
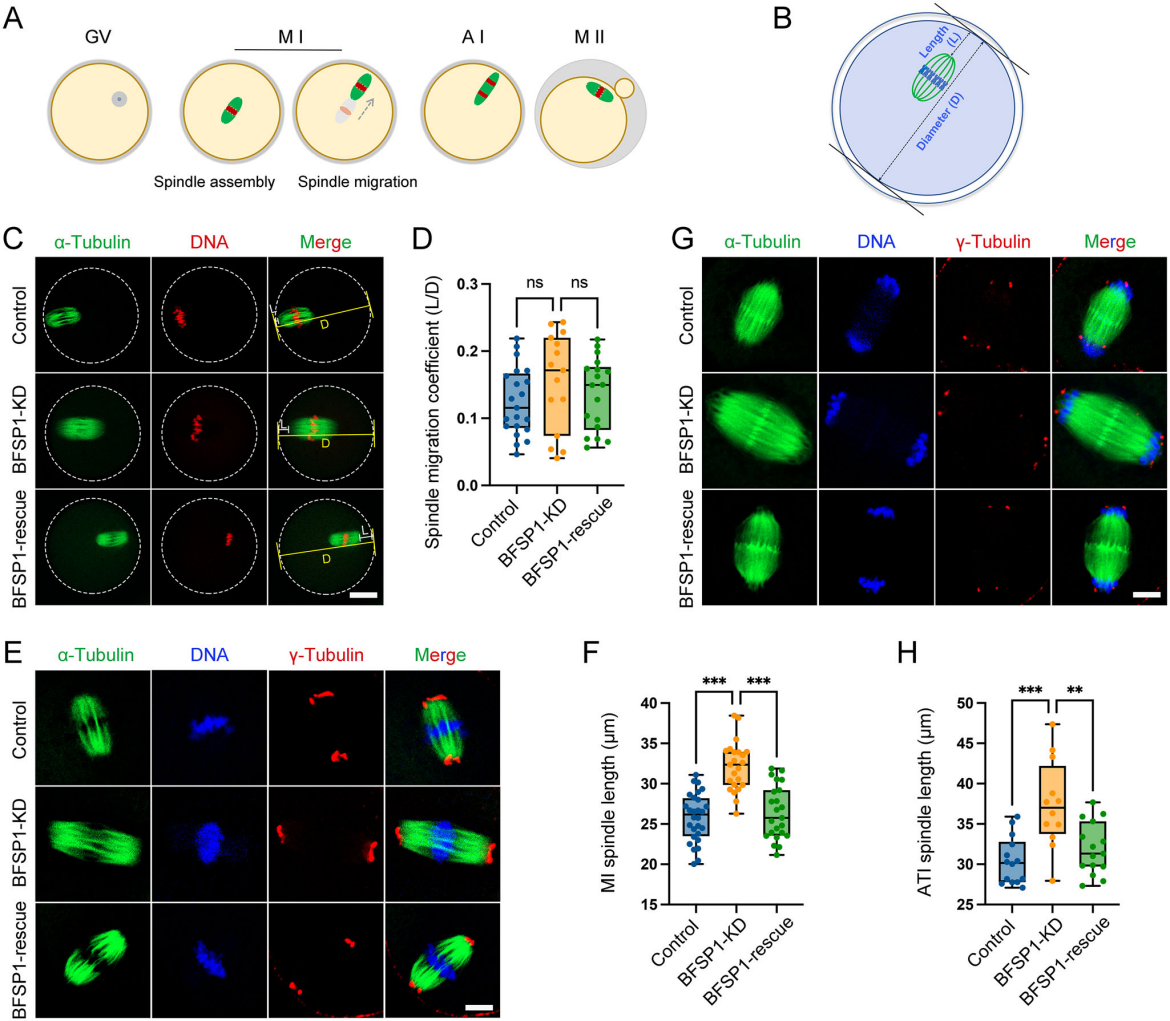
BFSP1 depletion compromises the spindle dynamics in oocytes. A) Schematic representation of spindle migration and asymmetric division during mouse oocyte meiosis. B) Schematic representation of spindle migration coefficient. L indicates the distance from the proximal spindle pole to the cortex, and D indicates the diameter of the oocyte. L/D indicates the spindle migration coefficient. C) Representative images of spindle migration in control, BFSP1‐KD, and BFSP1 rescue oocytes at 9 h post‐GV. White line indicates the distance from the proximal spindle pole to the cortex, and yellow line indicates the diameter of the oocyte. Scale bar, 20 µm. D) Spindle migration coefficient was calculated in control (*n* = 21), BFSP1‐KD (*n* = 15), and BFSP1‐rescue (*n* = 18) oocytes. E) Representative images of spindle length in control, BFSP1‐KD, and BFSP1‐rescue oocytes at M I stage. Oocytes were immunostained for α‐tubulin and γ‐tubulin. Scale bar, 15 µm. F) Spindle length was measured between two spindle poles in control (*n* = 28), BFSP1‐KD (*n* = 23), and BFSP1‐rescue (*n* = 23) oocytes at M I stage. G) Representative images of spindle length in control, BFSP1‐KD, and BFSP1‐rescue oocytes at AT I (anaphase/telophase I) stage (in vitro culture for 9 h 45 min post‐GV). Oocytes were immunostained for α‐tubulin and γ‐tubulin. Scale bar, 15 µm. H) Spindle length was measured between two spindle poles in control (*n* = 14), BFSP1‐KD (*n* = 12), and BFSP1‐rescue (*n* = 15) oocytes at AT I stage. Data in (D), (F), and (H) were expressed as mean ± SD of at least three independent experiments. ^**^ *P* < 0.01; ^***^ *P* < 0.001; ns, no significance.

Oocyte symmetric division is usually induced by two causes. One is the failure of spindle migration from oocyte center to the cortex,^[^ ^34^, ^35^ ^]^ and another is the elongation of spindle axis.^[^ ^36^ ^]^ To decipher how BFSP1 depletion breaks the oocyte asymmetric division, we first of all tested whether BFSP1 depletion affects the spindle migration (**Figure** **3**A). We defined the distance from spindle pole to the oocyte cortex as L and the oocyte diameter as D. The ratio of L to D was then used to represent the spindle migration coefficient in oocytes (Figure 3B). Fluorescence imaging combined with quantitative analysis displayed that spindle migration coefficient in BFSP1‐depleted oocytes was comparable to the control and BFSP1‐rescued oocytes (Figure 3C,D), implying that BFSP1 is not involved in the spindle migration. We thus quantified the length of spindle axis in M I and anaphase/telophase I (AT I) oocytes. We found that the spindle length in M I oocytes after BFSP1 depletion was dramatically longer than that in controls, while it restored to a normal level following expression of exogenous BFSP1‐6×HA (Figure 3E,F). Importantly, the elongation of the spindle lasted to AT I stage just prior to division in BFSP1‐depleted oocytes (Figure 3G,H). Therefore, our findings indicate that BFSP1 might maintain the asymmetric division of mouse oocytes via regulating the spindle length instead of spindle migration.

### 2.4 MAP1B is a Binding Partner of BFSP1 in Oocytes

**Figure 4:**
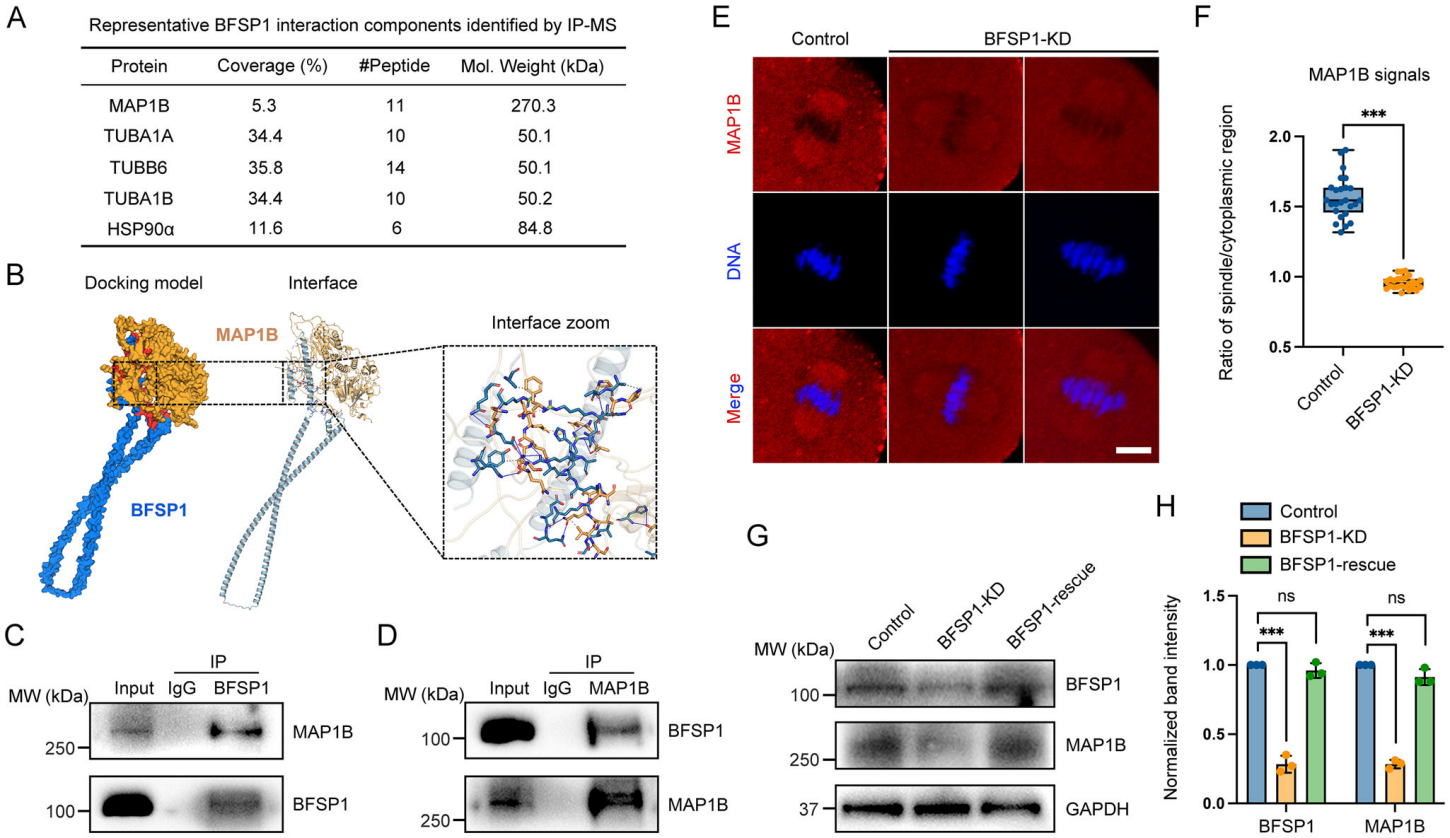
BFSP1 interacts with MAP1B and affects its protein stability. A) Identification of BFSP1 binding proteins by IP/MS analysis. Protein name, coverage percentage, the number of identified peptides, and molecular weight were shown in the table. B) Representation of the 3D structure and predicted interaction of mouse BFSP1 and MAP1B using AlphaFold databank by HDOCK server. C) Co‐IP using anti‐BFSP1 antibody followed by immunoblotting analysis with anti‐MAP1B and anti‐BFSP1 antibodies. D) Co‐IP using anti‐MAP1B antibody followed by immunoblotting analysis with anti‐BFSP1 and anti‐MAP1B antibodies. E) Representative images of MAP1B in control and BFSP1‐KD oocytes. Scale bar, 10 µm. F) The ratio of MAP1B fluorescence intensity in the spindle region to the cytoplasmic region was measured in control and BFSP1‐KD oocytes. G) Protein levels of MAP1B in control, BFSP1‐KD, and BFSP1‐rescue oocytes as assessed by immunoblotting analysis. The band intensity of BFSP1 and MAP1B was normalized with that of GAPDH. H) The band intensities of BFSP1 and MAP1B in the blots were normalized with that of GAPDH. Data in (F) were expressed as mean ± SD, and (H) were expressed as mean ± SEM of at least three independent experiments. ^***^ *P* < 0.001; ns, no significance.

To unveil the molecular mechanisms by which BFSP1 participates in the oocyte asymmetric division, we combined the immunoprecipitation (IP) with mass spectrometry (MS) to identify the binding proteins of BFSP1. In the MS data from BFSP1 precipitate, we discovered numerous microtubule proteins such as Tuba1a, Tuba1b, and Tubb6 (**Figure** **4**A), which confirmed our above observations about the spindle‐like localization pattern of BFSP1. This also suggests that BFSP1 may collaborate with cytoskeletal proteins to take part in oocyte meiosis. Among the top list of MS candidates, we noticed microtubule‐associated protein 1b (MAP1B) that has been implicated in regulating microtubule stability in mitotic cells^[^ ^37^, ^38^, ^39^, ^40^ ^]^ (Figure 4A). We applied AlphaFold database to predict the 3D protein structures of BFSP1 and MAP1B,^[^ ^41^, ^42^ ^]^ and docked these two proteins by HDOCK server to simulate their interaction.^[^ ^43^, ^44^, ^45^, ^46^, ^47^ ^]^ The docking score of the docking model was −216.54, and the confidence score was 0.79, indicative of a high possibility of interaction between BFSP1 and MAP1B (Figure 4B). In addition, we verified this interaction by conducting co‐IP experiments. Immunoblotting analysis revealed that MAP1B was present in the BFSP1 precipitate, and reciprocally BFSP1 was observed in the MAP1B precipitate, confirming the binding of MAP1B to BFSP1 (Figure 4C,D). We further investigated how BFSP1 impacts MAP1B dynamics in oocytes. By antibody staining and expression of exogenous MAP1B‐EGFP, we found that MAP1B accumulated on the spindle apparatus at M I and M II stages, similar as BFSP1 (Figure S4A,B, Supporting Information). Depletion of BFSP1 remarkably decreased the MAP1B signals in oocytes as assessed by fluorescence imaging and intensity quantification (Figure 4E,F). Consistent with this observation, immunoblotting results showed that the protein level of MAP1B considerable declined in BFSP1‐depleted oocytes (Figure 4G,H). Of note, the reduced MAP1B could be restored by expression of exogenous BFSP1‐6×HA (Figure 4G,H). Taken together, these data illustrate that BFSP1 binds to MAP1B and is required for its protein stability.

### 2.5 Depletion of MAP1B Causes Elongated Spindle and Symmetric Division in Oocytes

**Figure 5:**
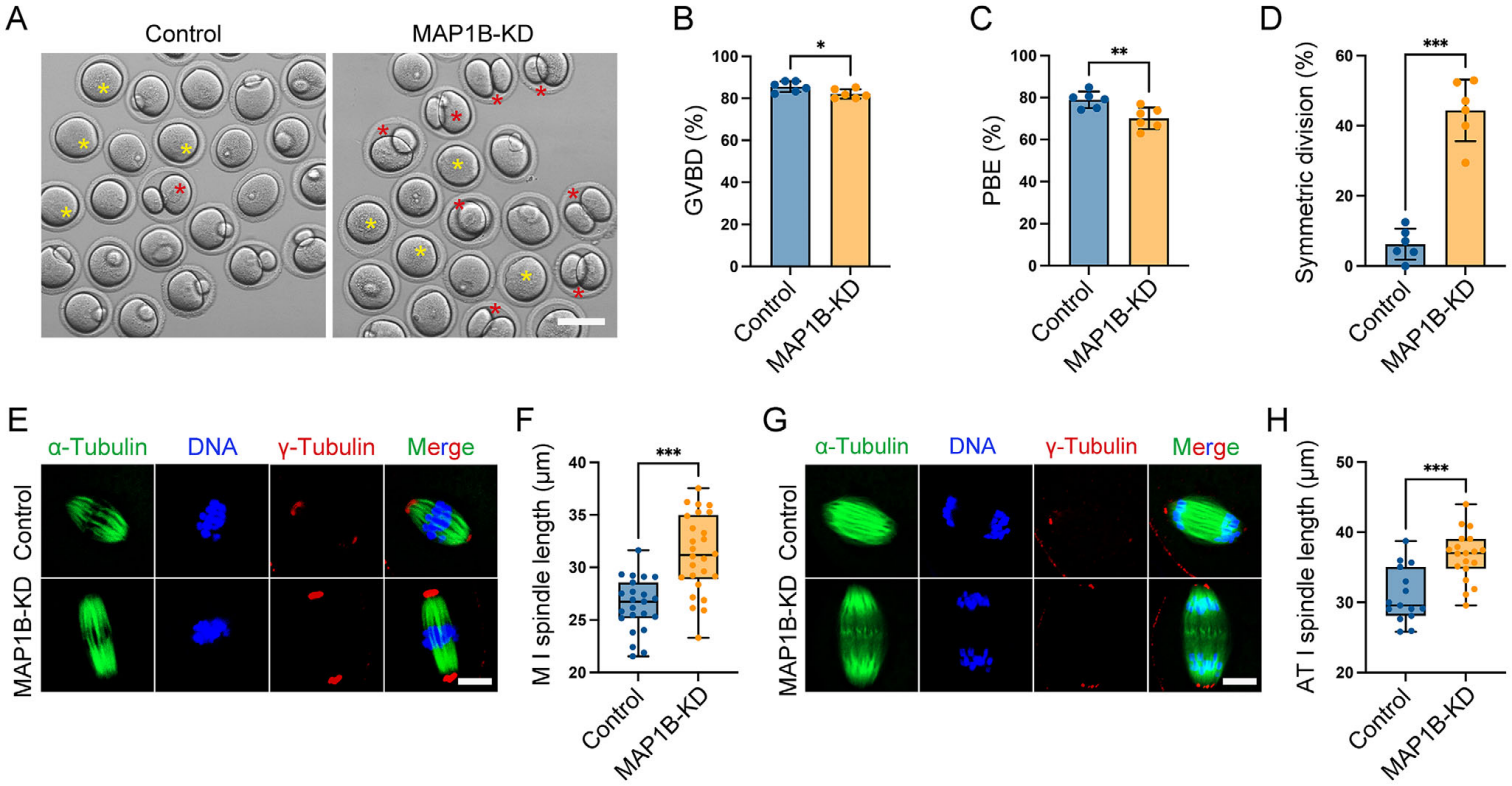
MAP1B depletion impairs the oocyte meiotic maturation and spindle length control. A) Representative images of oocytes at M II stage in control and MAP1B‐KD groups. Yellow asterisks indicate oocytes that failed to extrude the first polar body, and red asterisks indicate oocytes with symmetric division. Scale bar, 80 µm. B) The GVBD rate was quantified in control (*n* = 202) and MAP1B‐KD (*n* = 189) oocytes. C) The PBE rate was quantified in control (*n* = 202) and MAP1B‐KD (*n* = 189) oocytes. D) The rate of symmetric division was quantified in control (*n* = 202) and MAP1B‐KD (*n* = 189) oocytes. E) Representative images of spindle length in control and MAP1B‐KD oocytes at M I stage. Oocytes were immunostained for α‐tubulin and γ‐tubulin. Scale bar, 15 µm. F) Spindle length was measured between two spindle poles in control (*n* = 23) and MAP1B‐KD (*n* = 26) oocytes at M I stage. G) Representative images of spindle length in control and MAP1B‐KD oocytes at AT I stage. Oocytes were immunostained for α‐tubulin and γ‐tubulin. Scale bar, 15 µm. H) Spindle length was measured between two spindle poles in control (*n* = 15) and MAP1B‐KD (*n* = 19) oocytes at AT I stage. Data in (B), (C), and (D) were expressed as mean ± SEM, and (F) and (H) were expressed as mean ± SD of at least three independent experiments. ^*^ *P* < 0.05; ^**^ *P* < 0.01; ^***^ *P* < 0.001.

Although it has been reported that MAP1B is involved in the microtubule dynamics in mitotic cells,^[^ ^48^ ^]^ its role during oocyte meiosis remains unknown. Knockdown of MAP1B by microinjection of gene‐targeted siRNA significantly decreased the transcript and protein levels of MAP1B in oocytes (Figure S5, Supporting Information). Observation of meiotic progression of MAP1B‐depletion oocytes showed a slight decrease in the rate of GVBD (**Figure** **5**B). More importantly, the depletion of MAP1B lowered the percentage of PBE, but increased the proportion of symmetric division compared to the controls (Figure 5A–D), in line with the meiotic defects in BFSP1‐depleted oocytes. We further evaluated the effect of MAP1B depletion on the spindle length. Compared with the controls, the spindle length in MAP1B‐depleted oocytes at both M I and AT I stages was substantially longer (Figure 5E–H), phenocopying the BFSP1‐depleted oocytes. The docking model for BFSP1 and MAP1B interactions identified three key domains in MAP1B sequence that is required for its binding to BFSP1. We then constructed a MAP1B mutant with deletion of 1118–1279aa/1554‐1593aa/2089‐2093aa (Figure S6A, Supporting Information), and expressed it in oocytes to observe the consequences. It was shown that mutation of MAP1B did not influence the GVBD and PBE, but caused a high percentage of large polar bodies (Figure S6B–E, Supporting Information). Also, mutation of MAP1B induced the formation of elongated spindles in oocytes at both M I and AT I stages (Figure S6F–I, Supporting Information). In conclusion, these findings suggest that MAP1B might mediate the role of BFSP1 in the asymmetric division of oocytes.

### 2.6 Expression of MAP1B in BFSP1‐Depleted Oocytes Inhibits the Occurrence of Symmetric Division

**Figure 6:**
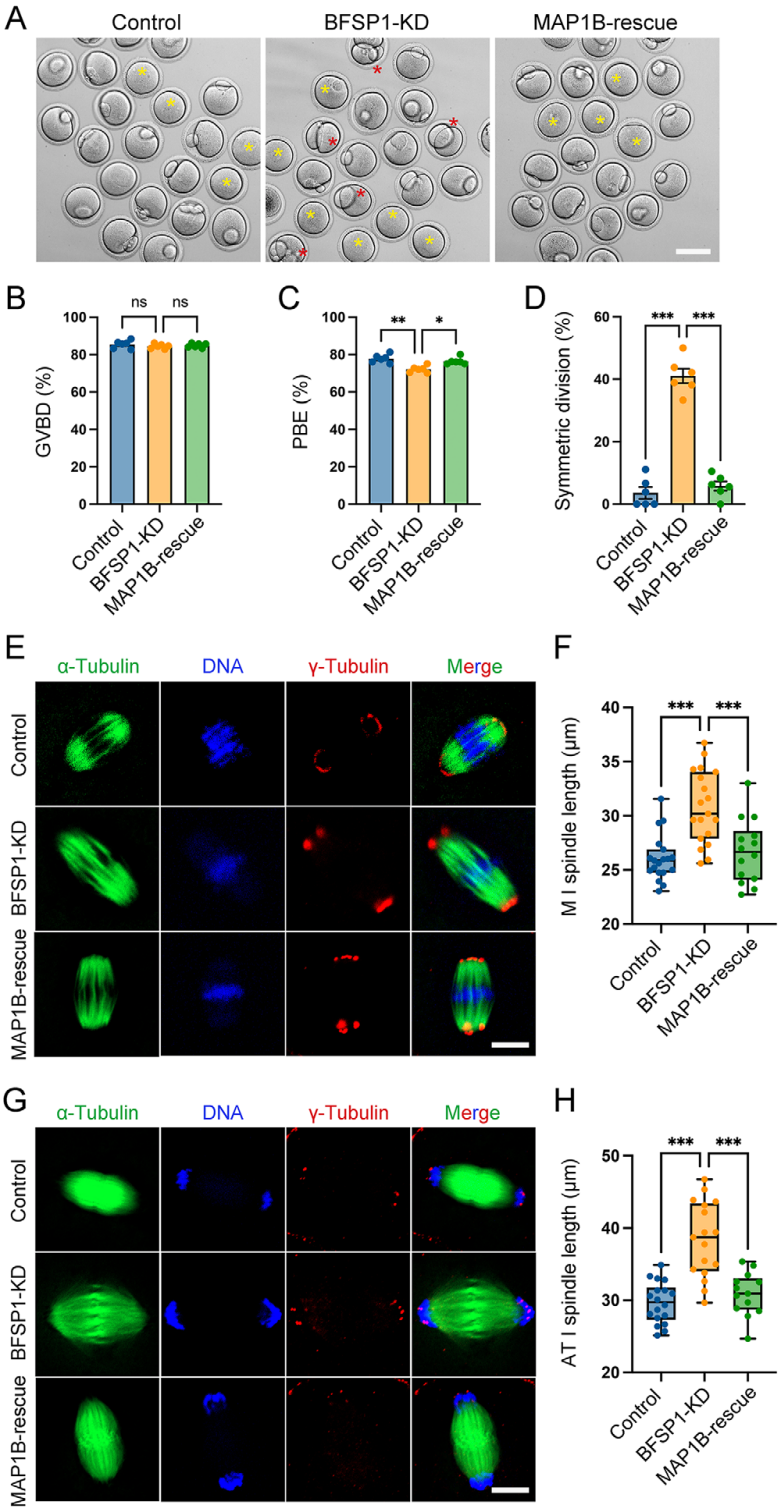
Restored MAP1B protein levels mitigate the meiotic defects induced in BFSP1 depleted‐oocytes. A) Representative images of oocytes at M II stage in control, BFSP1‐KD, and MAP1B‐rescue groups. For the rescue experiment, MAP1B‐EGFP mRNA was microinjected to GV oocytes 20 h after microinjection of BFSP1 siRNAs. Yellow asterisks indicate oocytes that failed to extrude the first polar body, and red asterisks indicate oocytes with symmetric division. Scale bar, 80 µm. B) The GVBD rate was quantified in control (*n* = 180), BFSP1‐KD (*n* = 174), and MAP1B‐rescue (*n* = 185) oocytes. C) The PBE rate was quantified in control (*n* = 180), BFSP1‐KD (*n* = 174), and MAP1B‐rescue (*n* = 185) oocytes. D) The rate of symmetric division was quantified in control (*n* = 180), BFSP1‐KD (*n* = 174), and MAP1B‐rescue (*n* = 185) oocytes. E) Representative images of spindle length in control, BFSP1‐KD, and MAP1B‐rescue oocytes at M I stage. Oocytes were immunostained for α‐tubulin and γ‐tubulin. Scale bar, 15 µm. F) Spindle length was measured between two spindle poles in control (*n* = 19), BFSP1‐KD (*n* = 19), and MAP1B‐rescue (*n* = 14) oocytes at M I stage. G) Representative images of spindle length in control, BFSP1‐KD, and MAP1B‐rescue oocytes at AT I stage. Oocytes were immunostained for α‐tubulin and γ‐tubulin. Scale bar, 15 µm. H) Spindle length was measured between two spindle poles in control (*n* = 18), BFSP1‐KD (*n* = 17), and MAP1B‐rescue (*n* = 13) oocytes at AT I stage. Data in (B), (C), and (D) were expressed as mean ± SEM, and (F) and (H) were expressed as mean ± SD of at least three independent experiments. ^*^ *P* < 0.05; ^**^ *P* < 0.01; ^***^ *P* < 0.001; ns, no significance.

To test whether MAP1B is the downstream effector of BFSP1, we expressed exogenous MAP1B‐EGFP in BFSP1‐depleted oocytes to examine the phenotype. Immunoblotting data verified that MAP1B levels were recovered in BFSP1‐depleted oocytes after expression of exogenous MAP1B‐EGFP (Figure S7, Supporting Information). Consistent with above observations, GVBD was not affected by BFSP1 depletion, but PBE rate showed a decrease in comparison with the controls (**Figure** **6**A–C). Importantly, expression of MAP1B elevated the PBE rate in BFSP1‐depleted oocytes (Figure 6A,C). In the meantime, the high proportion of symmetric division occurring in BFSP1‐depleted oocytes was also lowered by expression of MAP1B (Figure 6A,D), suggesting that restoration of MAP1B could attenuate the meiotic defects induced by BFSP1 loss‐of‐function. Moreover, we measured the spindle length in both M I and AT I oocytes, and showed that expression of MAP1B shortened the spindle axis in BFSP1‐depleted oocytes (Figure 6E–H), highlighting the role of MAP1B in mediating the BFSP1 function in oocytes.

### 2.7 BFSP1 Recruits HSP90α to Maintain MAP1B Protein Stability

**Figure 7:**
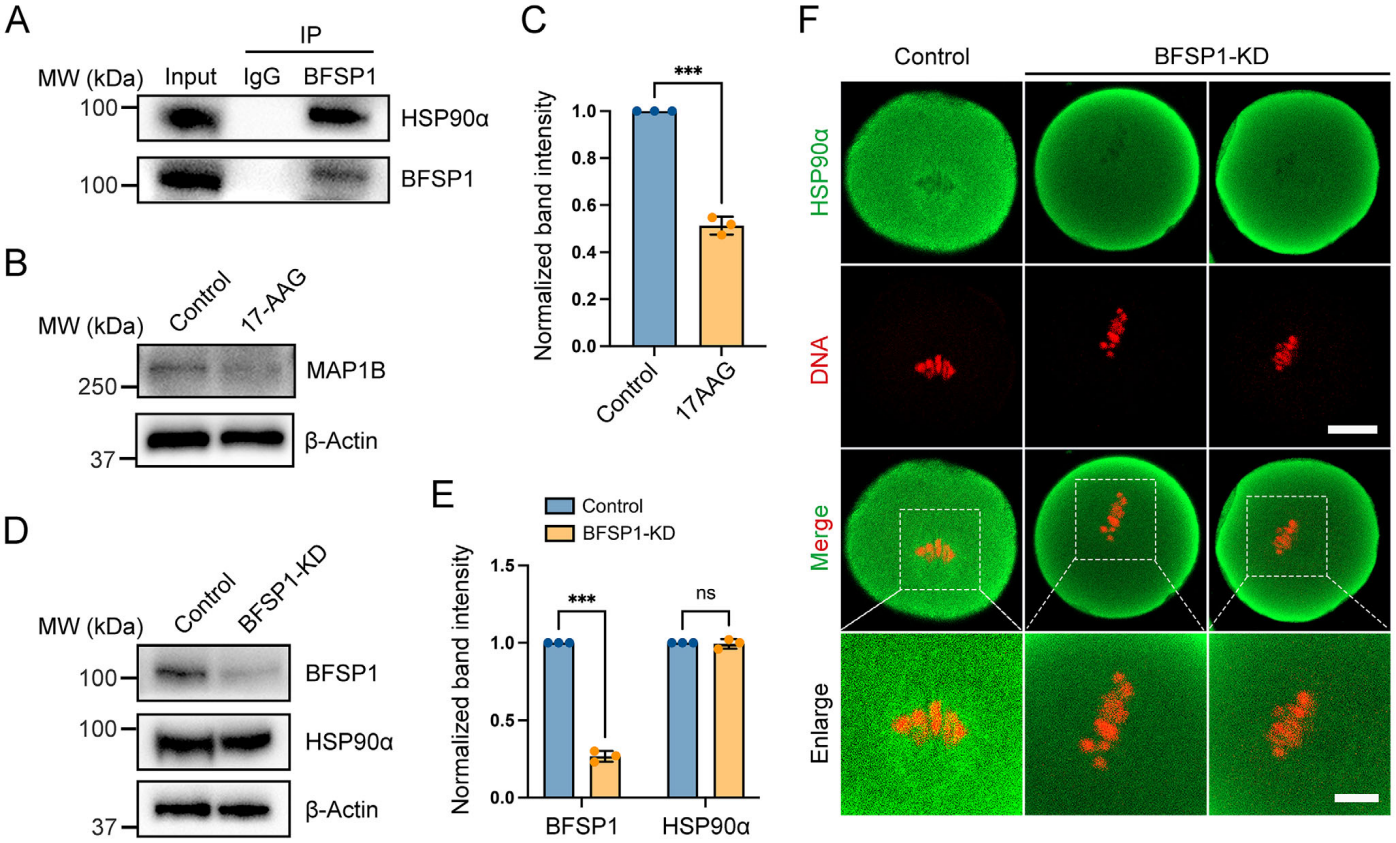
BFSP1 maintains MAP1B protein levels by recruiting HSP90α. A) Co‐IP using anti‐BFSP1 antibody followed by immunoblotting analysis with anti‐HSP90α and anti‐BFSP1 antibodies. B) Protein levels of MAP1B in control and 17‐AAG‐treated oocytes as assessed by immunoblotting analysis. C) The band intensity of MAP1B in the blots was normalized with that of β‐Actin. D) Protein levels of HSP90α in control and BFSP1‐KD oocytes as assessed by immunoblotting analysis. E) The band intensities of BFSP1 and HSP90α in the blots were normalized with that of β‐Actin. F) Representative images of HSP90α localization in the spindle region in control and BFSP1‐KD oocytes. Scale bars, 20 µm, 10 µm. Data in (C) and (E) were expressed as mean ± SEM of at least three independent experiments. ^***^ *P* < 0.001; ns, no significance.

We further explored the underlying mechanism regarding how BFSP1 regulates MAP1B protein levels. Revisiting the MS data, we found that a molecular chaperone heat shock protein 90α (HSP90α) was also a potential binding protein of BFSP1. Their interaction was confirmed by the co‐IP experiment performed with BFSP1 antibody (**Figure** **7**A). Given that previous report has shown that deletion of HSP90α promotes MAP1B degradation in the retina,^[^ ^39^ ^]^ we next asked whether HSP90α is responsible for MAP1B stability in oocytes. 17‐allylamino‐demethoxygeldanamycin (17‐AAG), a potent HSP90α inhibitor, was used to treat oocytes to inhibit HSP90α. Immunoblotting analysis displayed that protein levels of MAP1B were significantly decreased in 17‐AAG‐treated oocytes compared to the control oocytes (Figure 7B,C), indicating that HSP90α is also a molecular chaperon for MAP1B in oocytes. Moreover, we observed the HSP90α dynamics in BFSP1‐depleted oocytes. The total protein levels of HSP90α were not changed by BFSP1 depletion as evaluated by immunoblotting (Figure 7D,E). However, immunostaining results showed that HSP90α accumulated on the spindle apparatus in control oocytes (Figure S8, Supporting Information; Figure 7F), but disappeared in BFSP1‐depleted oocytes (Figure 7F), suggesting that BFSP1 is required for recruitment of HSP90α to the spindle for stabilizing MAP1B.

## 3 Discussion

The cytoskeleton has been implicated in diverse cellular events during oocyte meiosis, including the formation of spindle apparatus, distribution of organelles, and asymmetric division.^[^ ^33^, ^49^, ^50^ ^]^ Among them, microtubules and microfilaments are well studied and function differentially in these events,^[^ ^51^, ^52^, ^53^ ^]^ while the involvement of intermediate filaments and associated proteins remains largely unknown. Interestingly, a recent work has shown the interactions and cooperative mechanisms between microtubules and microfilaments. Actin is found in close association with microtubules in the spindle and promotes chromosome segregation fidelity during meiosis I and II in mammalian oocytes.^[^ ^54^ ^]^ In the present study, our findings provide additional evidence to illustrate that intermediate filament protein interacts with microtubules to drive the asymmetric division of mouse oocytes.

Intermediate filament proteins BFSP1 and BFSP2 are mainly found in lens fiber cells to assemble the beaded filament structure, a classical filamentous network beneath the plasma membrane that spans the cytoplasm in a loosely arranged meshwork.^[^ ^24^, ^55^, ^56^, ^57^, ^58^ ^]^ In addition, both proteins are also expressed in mouse lens epithelium to form a tubular structure, which co‐localizes with actin instead of γ‐tubulin and α‐tubulin.^[^ ^55^ ^]^ Knockout or mutation of BFSP1 leads to reduced lens transparency and cataract formation.^[^ ^27^, ^28^ ^]^ Strikingly, our data revealed that BFSP1 was present in oocytes at different developmental stages during meiotic maturation as well. In particular, it accumulated on the microtubule fibers of the spindle, suggesting that BFSP1 may play a microtubule dynamics‐related role during meiosis.

Our loss‐of‐function study of BFSP1 in oocytes showed that occurrence of symmetric division is the major meiotic defect. In contrast to symmetric division in mitosis, mammalian oocyte undergoes asymmetric cell division to produce a large oocyte and a tiny polar body,^[^ ^59^, ^60^ ^]^ which is critical for the retention of maternal materials in oocytes for subsequent fertilization and early embryo development.^[^ ^32^, ^61^ ^]^ In addition, the small size of polar body is also required to avoid the competition between symmetrically divided cells during fertilization. Due to the lack of microvillosities and reduced cell surface, the polar body cannot support the sperm binding and be fertilized.^[^ ^60^, ^62^ ^]^ A variety of molecules have been reported to play critical roles in the oocyte asymmetric division, including Rab14, FHOD1, Filamin A, Capping protein, and Nampt.^[^ ^34^, ^36^, ^63^, ^64^, ^65^ ^]^ Furthermore, previous studies have indicated that spindle migration failure is a primary cause of symmetric division in oocytes.^[^ ^34^, ^35^ ^]^ Meanwhile, spindle elongation can also impair oocyte asymmetry. Elongated spindles slow down the movement of midzones and induce cortical furrowing deep within the oocyte before protrusions form, thus resulting in larger polar body being cleaved off.^[^ ^36^ ^]^ These facts prompted us to further investigate the impact of BFSP1 on the spindle migration and spindle length. Our results corroborated that absence of BFSP1 in oocytes did not result in spindle migration failure but caused elongation of the spindle, revealing that BFSP1 takes part in the asymmetric division of oocytes by regulating spindle length.

Another important finding in our study is that we elucidated the molecular mechanism by which BFSP1 functions in the meiotic asymmetric division. By IP experiment combined with MS analysis, we identified a large number of cytoskeleton‐associated proteins as the potential binding candidates for BFSP1, especially microtubule‐associated proteins. This is consistent with our observation that the localization and function of BFSP1 in oocytes is related to the microtubules. Among the candidates, we noticed a microtubule‐associated protein MAP1B, because previous studies have shown that MAP1B co‐localizes with microtubules in neuronal cells,^[^ ^40^, ^66^, ^67^, ^68^, ^69^ ^]^ and exerts functions in microtubule stability to promote axonal elongation, and neuronal migration, and axonal guidance.^[^ ^70^, ^71^ ^]^ In addition, purified recombinant MAP1B facilitates the formation of shorter microtubules by accelerating their assembly.^[^ ^72^ ^]^ In our study, we evidenced that MAP1B also exhibited a spindle‐like localization pattern in oocytes, and knockdown or mutation of MAP1B phenocopied the meiotic defects induced by BFSP1 depletion, resulting in elongated spindles and symmetric division of oocytes. Moreover, we manifested that BFSP1 depletion led to the reduced protein level of MAP1B, and expression of MAP1B in BFSP1‐depleted oocytes recovered the spindle length and asymmetric division, which indicates that MAP1B is the downstream target of BFSP1 that mediates its function during oocyte meiosis.

Interestingly, we also found a heat shock protein family member HSP90α in our MS data. HSP90α is a highly conserved and ubiquitously expressed molecular chaperone protein that is involved in protein folding, stability, and trafficking.^[^ ^73^ ^]^ It has been reported that inhibition of HSP90α activity with its specific inhibitor 17‐allylamino‐demethoxygeldanamycin (17‐AAG) leads to the symmetric division in mouse oocytes, resulting in the formation of large polar bodies.^[^ ^74^ ^]^ Besides, in HSP90α‐deficient mice, the absence of HSP90α in the retina promotes MAP1B degradation by inducing ubiquitination.^[^ ^39^ ^]^ Similarly, our results showed that BFSP1 is required for spindle localization of HSP90α to stabilize MAP1B during oocyte meiosis.

In conclusion, our study uncovers a unique role of intermediate filament protein BFSP1 as a spindle regulator to maintain the asymmetric division of mouse oocytes. BFSP1 accumulates on the spindle apparatus to provide a scaffold platform for the docking of molecular chaperone HSP90α with its target MAP1B to protect the protein stability of MAP1B, thereby regulating proper microtubule dynamics and spindle length (Figure S9, Supporting Information). These findings extend our understanding about the cooperative interactions among different cytoskeletal‐associated proteins in driving the oocyte meiotic progression. However, in the current study, we did not provide direct evidence demonstrating whether BFSP1 modulates the oocyte asymmetric division as a component of intermediate filaments or not. It would be interesting to determine the assembly of intermediate filaments in oocytes in future investigations.

## 4 Experimental Section

### Animals

All experiments in this study were performed using 6–8‐week‐old female Institute of Cancer Research (ICR) mice, and mouse protocols and experimental procedures were approved by the Animal Research Institute Committee of Nanjing Agricultural University, China.

### Antibodies

Rabbit polyclonal anti‐BFSP1 antibody (Cat# A3764) and rabbit monoclonal anti‐Myc antibody (Cat# AE070) were purchased from Abclonal (Wuhan, China); rabbit polyclonal anti‐MAP1B antibody (Cat# 21633‐1‐AP), rabbit polyclonal anti‐HSP90α antibody (Cat# 13171‐1‐AP), mouse monoclonal anti‐β‐Actin antibody (Cat# 66009‐1‐lg), rabbit polyclonal anti‐HA antibody (Cat# 51064‐2‐AP), and mouse monoclonal anti‐GAPDH antibody (Cat# 60004‐1‐lg) were purchased from Proteintech (Rosemont, IL, USA); mouse monoclonal anti‐α‐Tubulin‐FITC antibody (Cat# F2168) was purchased from Sigma–Aldrich (St. Louis, MO, USA); rabbit monoclonal anti‐Vinculin antibody (Cat# CY5164) was purchased from Always (Shanghai, China).

### Oocyte Collection and In Vitro Culture

Fully‐grown oocytes arrested at GV stage were collected from the ovaries of female ICR mice in M2 medium (Luanchuang Lifetech, Nanjing, China; Cat# M01‐B), and then cultured in M16 medium (Luanchuang Lifetech; Cat# M02‐B) under liquid paraffin oil at 37 °C in an atmosphere of 5% CO_2_ incubator until they reached the desired stage.

### siRNA Knockdown

Three BFSP1‐targeting siRNA oligos or Three MAP1B siRNA oligos (GenePharma, Shanghai, China) were mixed and diluted with water to provide a working concentration of 25 µm, and then ≈5 to 10 pl of oligos were microinjected into the cytoplasm of fully grown mouse GV oocytes using a Narishige microinjector. A non‐targeting siRNA oligo was injected as a control. To facilitate the degradation of mRNA by siRNA, mouse oocytes were arrested at GV stage in M16 medium containing 50 µm 3‐Isobutyl‐1‐methylxanthine (IBMX) for 20 h and then transferred to IBMX‐free M16 medium to resume the meiosis for subsequent experiments. The siRNA sequences were listed in Table S1 (Supporting Information).

### mRNA Construct and In Vitro Transcription

PcDNA3.1/BFSP1‐6×Myc was purchased from Tsingke (Beijing, China); pcDNA3.1/MAP1B‐EGFP and pcDNA3.1/BFSP1‐6×HA were purchased from Gene Create (Wuhan, China). Capped mRNA was synthesized from linearized plasmid using T7 High Yield RNA Transcription kit (Vazyme, Nanjing, China; Cat# DD4201), and purified with MEGAclear kit (ThermoFisher Scientific, Waltham, MA, USA; Cat# AM1908). Typically, 10–12 pl of 0.5–1.0 µg µl^−1^ mRNA was injected into oocytes and then arrested at GV stage in M16 medium containing 50 µm IBMX for 4 h, allowing enough time for translation, followed by releasing into IBMX‐free M16 medium for further study.

### RNA Isolation and Quantitative Real‐Time PCR

Total RNA was extracted from a total of 30 oocytes using Dynabeads mRNA DIRECT Kit (ThermoFisher Scientific), and then reversed to cDNA and stored at −20 °C until use. Gene expression was determined by quantitative real‐time (RT)‐PCR using a ChamQ SYBR qPCR Master Mix (Vazyme, Nanjing, China). Each PCR reaction consisted of 10 µl of 2 × ChamQ SYBR qPCR Master Mix, 7 µl of water, 2 µl of cDNA sample, and 1 µl of gene‐specific primers, and was run in a Light Cycler instrument (ThermoFisher Scientific).

### Immunofluorescence Staining and Confocal Microscopy

Oocytes were fixed in 4% paraformaldehyde in PBS (pH 7.4) for 30 min and permeabilized in 0.5% Triton‐X‐100 for 20 min at room temperature. Oocytes were then blocked with 1% BSA‐supplemented PBS for 1 h and incubated with BFSP1 (1:100), MAP1B (1:100), α‐Tubulin‐FITC (1:500), HA (1:100), Myc (1:100), or HSP90α (1:100) antibodies at 4 °C overnight. After washing in PBST, oocytes were incubated with a corresponding secondary antibody for 1 h at room temperature, and then counterstained with 10 µg ml^−1^ Hoechst 33342 or propidium iodide (PI) for 10 min. Lastly, oocytes were mounted on glass slides and imaged by laser confocal microscope (LSM 900, Carl Zeiss, Germany). The quantification of fluorescence intensity was performed as we described previously.^[^ ^75^ ^]^ In brief, images from both control and treatment oocytes were acquired by confocal microscope using the same settings (identical laser power, pinhole, pixel dwell time). Image J (NIH, Bethesda, MD, USA) was then applied to define a region of interest (ROI) in the image, and the average fluorescence intensity per unit area within the ROI was determined. The average values of all measurements were used to compare the final average intensities between the control and treatment groups.

### Immunoprecipitation and Immunoblotting

Immunoprecipitation was carried out using 20 mouse ovaries according to the instruction for ProFound Mammalian CoImmunoprecipitation Kit (ThermoFisher Scientific). For immunoblotting, 150–200 oocytes were lysed in 4 × LDS sample buffer (ThermoFisher) containing protease inhibitor, and then separated on 10% Bis‐Tris precast gels and transferred onto PVDF membranes. The blots were blocked in TBST containing 5% low‐fat dry milk for 1 h at room temperature and then incubated with BFSP1 (1:1000), MAP1B (1:1000), GAPDH (1:5000), HA (1:5000), Myc (1:5000), HSP90α (1:2000), β‐Actin (1:5000), or Vinculin (1:5000) antibodies overnight at 4 °C. After three times of wash in TBST, the blots were incubated with HRP (horse radish peroxidase) conjugated secondary antibodies for 1 h at room temperature. Chemiluminescence signals were detected with ECL Plus (ThermoFisher Scientific) and protein bands were acquired by Tanon‐3900 Chemiluminescence Imaging System (Tanon, Beijing, China). Band intensities were quantified using Image J software and normalized to loading controls.

### Liquid Chromatography‐MS/MS and Data Analysis

The iTRAQ technology was carried out with the help of Gene Create Biolabs Inc. (Wuhan, China). For this purpose, total proteins were isolated from each biological sample. The protein concentration was measured using the Bradford method. Following digestion with Trypsin Gold (Promega, Madison, WI, USA), the peptides were dried, reconstituted in 0.5 m triethylammonium bicarbonate (TEAB) buffer (ThermoFisher Scientific), and then processed with 8‐plex iTRAQ reagent (ThermoFisher Scientific), according to the manufacturer's protocol.

The mixed peptides were fractionated using the Ultimate 3000 HPLC system (Thermo DINOEX, USA). Mass spectrometry data were generated using the TripleTOF 5600 + liquid mass spectrometry system (SCIEX, USA) coupled with the Eksigent nanoLC system (SCIEX). TripleTOF 5600plus liquid chromatography and mass spectrometry system (SCIEX) was used for mass spectrometry data acquisition.

The original MS/MS file data were analyzed by ProteinPilot Software v4.5, with the unused score ≥ 1.3 (corresponding to proteins identified with ≥ 95% confidence). An automatic decoy database search strategy was employed to estimate the false discovery rate (FDR) using the PSPEP (Proteomics System Performance Evaluation Pipeline Software, integrated with the ProteinPilot Software). Fold changes (FCs) were calculated as the average comparison pairs among biological replicates to determine differentially expressed proteins (DEPs). Proteins with an FC greater than 2 (upregulate ≥ 2.00 and down‐regulate ≤ 0.50) and a Q‐value less than 0.05 were considered significantly differentially expressed.

### Molecular Modeling

The 3D predictive structures of mouse wild‐type BFSP1 (A2AMT1) and MAP1B (P14873) were extracted from the AlphaFold databank. Protein–protein docking was performed by HDOCK server, and the docking model was displayed using pymol. To determine the reliability of the docking model, two factors need to be considered, including docking score and confidence score. The docking score reflects the binding affinity between the ligand and the receptor. A lower (more negative) score typically suggests a stronger binding interaction. The confidence score in docking refers to a numerical value that indicates the reliability or accuracy of a docking prediction. When the confidence score was above 0.7, the likelihood of binding was high, and when the confidence score was below 0.5, the likelihood of binding was considered low.

### In Vitro Fertilization

In vitro fertilization was carried out as we previously described.^[^ ^76^ ^]^ Briefly, sperm were released from the cauda epididymides of male mice (3–6‐month old) in human tubal fluid (HTF) medium and capacitated for 1 h at 37 °C in 5% CO_2_. 4 × 10^5^/ml capacitated sperm were then incubated with ovulated oocytes in 100 µl HTF for 6–8 h at 37 °C in a humidified atmosphere of 5% CO_2_. The presence of pronuclei was scored as successful fertilization.

### 17‐AGG Treatment

The 17‐AAG powder (MedChemExpress, HY‐10211; Monmouth Junction, NJ, USA) was dissolved in dimethyl sulfoxide (DMSO) to prepare a 10 mm stock solution, and then diluted to a working concentration of 2 µm with M16 medium. GV oocytes were treated with 17‐AAG in the M16 medium containing IBMX for 24 h. The concentration of DMSO in the medium was less than 0.1%.

### Statistical Analysis

All statistical data from at least three independent experiments were presented as mean ± SEM or SD unless otherwise stated, and the number of samples used in each group was labeled in parentheses as (n). Data were analyzed by a two‐tailed unpaired *t*‐test, which was provided by GraphPad Prism 10 statistical software. Data collection and analysis were not performed blinded to the conditions of the experiments. P < 0.05 was considered as statistical significance.

## Conflict of Interest

The authors declare no conflict of interest.

## Author Contributions

B.X. designed the research; Y.L., H.Z., W.Z., Y.M., and Y.Z. performed the experiments; Y.L., S.S., and B.X. analyzed the data; Y.L. and B.X. wrote the manuscript.

## Supporting information



Supporting Information

Supporting Dataset

## Data Availability

The data that support the findings of this study are available in the supplementary material of this article.
