# Supplementary material for: Intermediate Filament Protein BFSP1 Maintains Oocyte Asymmetric Division by Modulating Spindle Length
Source: Adv Sci (Weinh). 2025 May 11;12(28):2504066. doi: 10.1002/advs.202504066 (PMC12302563; doi:10.1002/advs.202504066)
Supplement: Supplementary file 1 — Supporting Information [file ADVS-12-2504066-s001.doc]

**Supplementary Information**

**Intermediate filament protein BFSP1 maintains oocyte asymmetric division by modulating spindle length**

Yu Li, Hanwen Zhang, Wenjun Zeng, Yilong Miao, Shaochen Sun, Yu Zhang, Bo Xiong


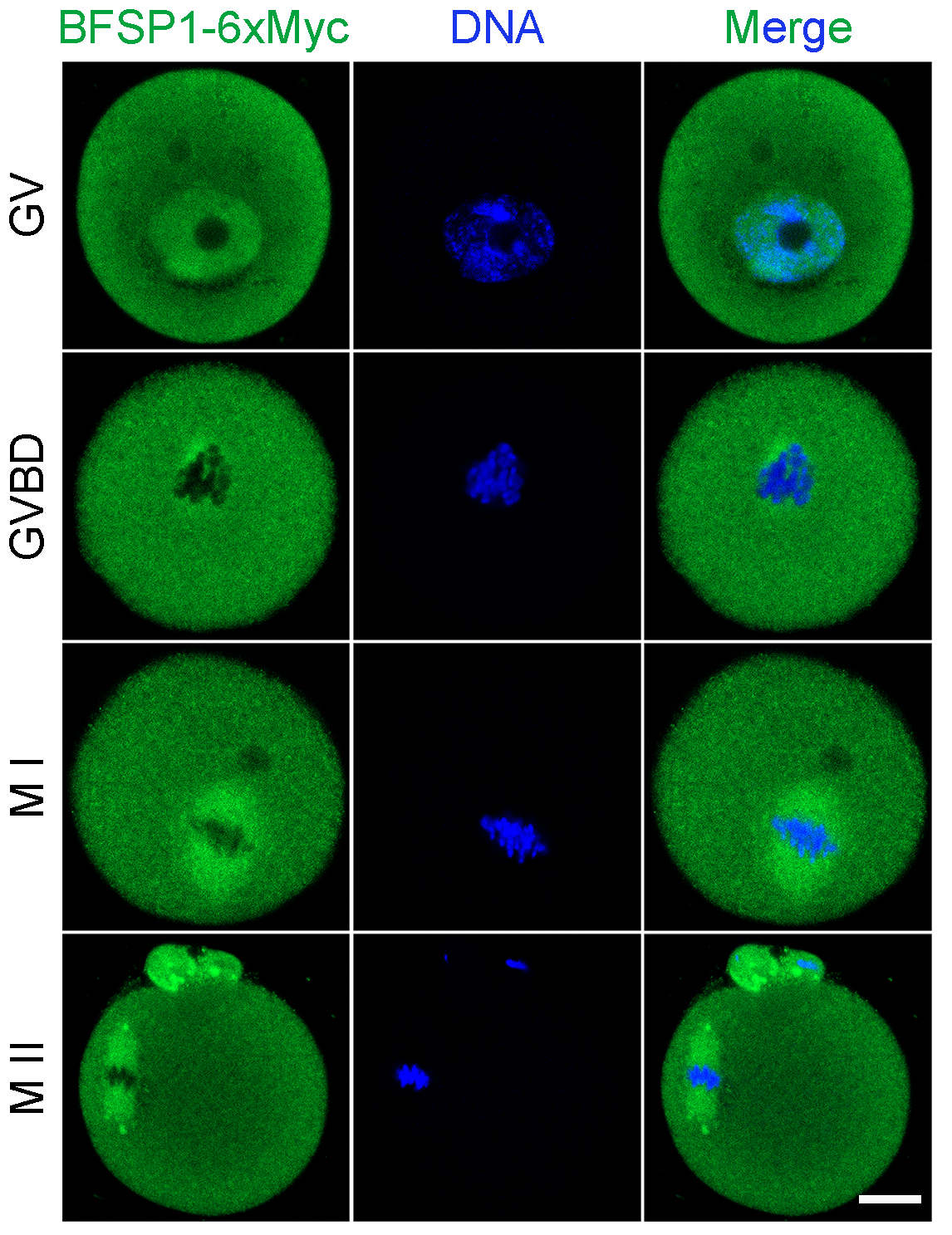


**Fig. S1. Localization of exogenous BFSP1-6×Myc in oocytes.** Representative images of BFSP1-6×Myc localization during oocyte meiotic maturation. Scale bar, 20 μm.


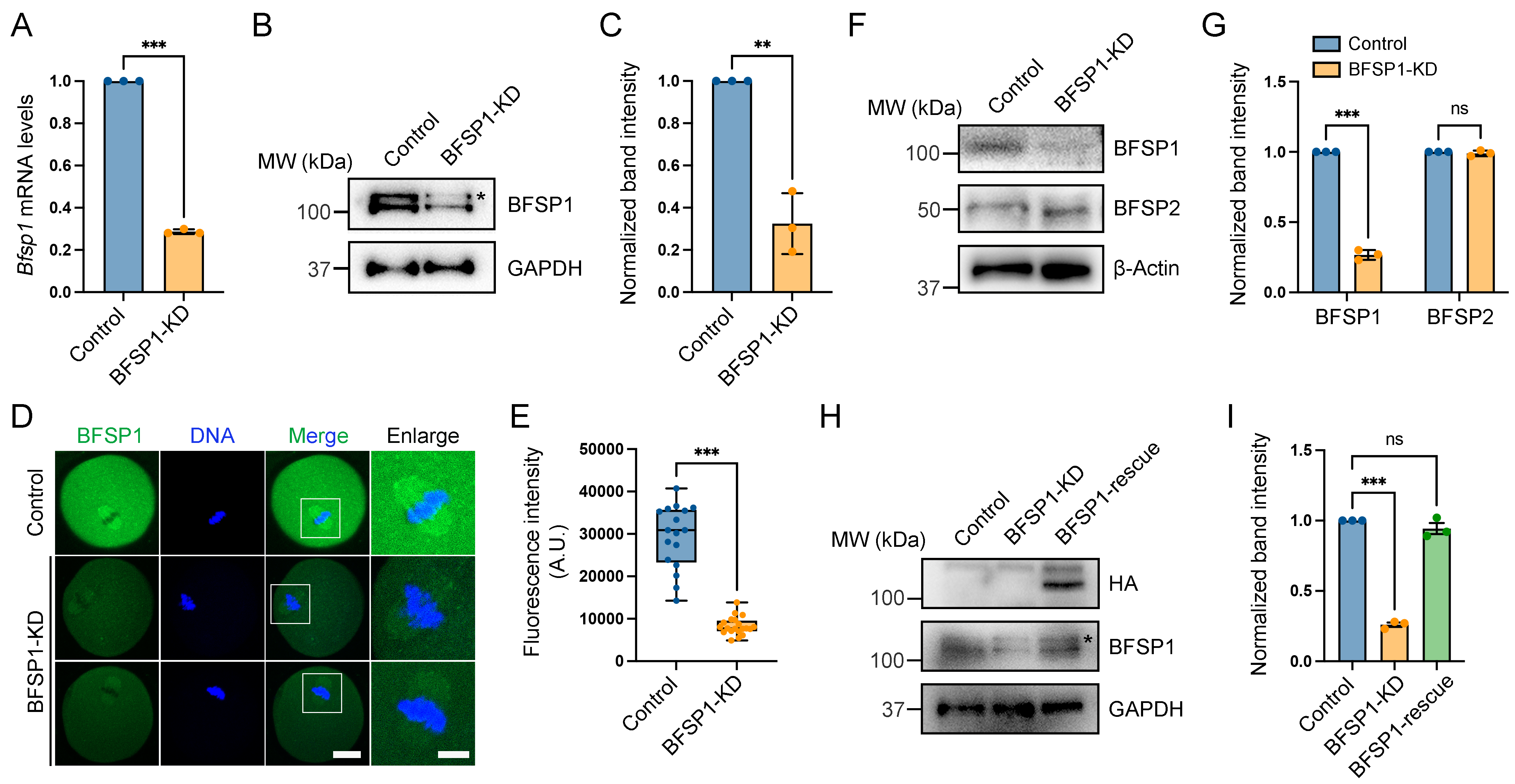


**Fig. S2. Knockdown and overexpression efficiency of BFSP1 in oocytes.** (A) mRNA levels of *Bfsp1* in control and BFSP1-KD oocytes as assessed by qRT-PCR. (B) Protein levels of BFSP1 in control and BFSP1-KD oocytes as assessed by immunoblotting analysis. Asterisk indicates the nonspecific bands. (C) The band intensity of BFSP1 in the blots was normalized with that of GAPDH. (D) Fluorescence signals of BFSP1 in control and BFSP1-KD oocytes as assessed by immunostaining. Scale bars, 20 μm, 10 μm. (E) Fluorescence intensity of BFSP1 signals was quantified in control (n = 17) and BFSP1-KD (n = 20) oocytes. (F) Protein levels of BFSP2 in control and BFSP1-KD oocytes as assessed by immunoblotting analysis. (G) The band intensities of BFSP1 and BFSP2 in the blots were normalized with that of β-Actin. (H) Protein levels of BFSP1 in control, BFSP1-KD, and BFSP1-rescue oocytes as assessed by immunoblotting analysis. Asterisk indicates the nonspecific bands. (I) The band intensity of BFSP1 in the blots was normalized with that of GAPDH. Data in (A), (C), (G) and (I) were expressed as mean ± SEM, and (E) were expressed as mean ± SD of at least three independent experiments. **P < 0.01; ***P < 0.001; ns, no significance.


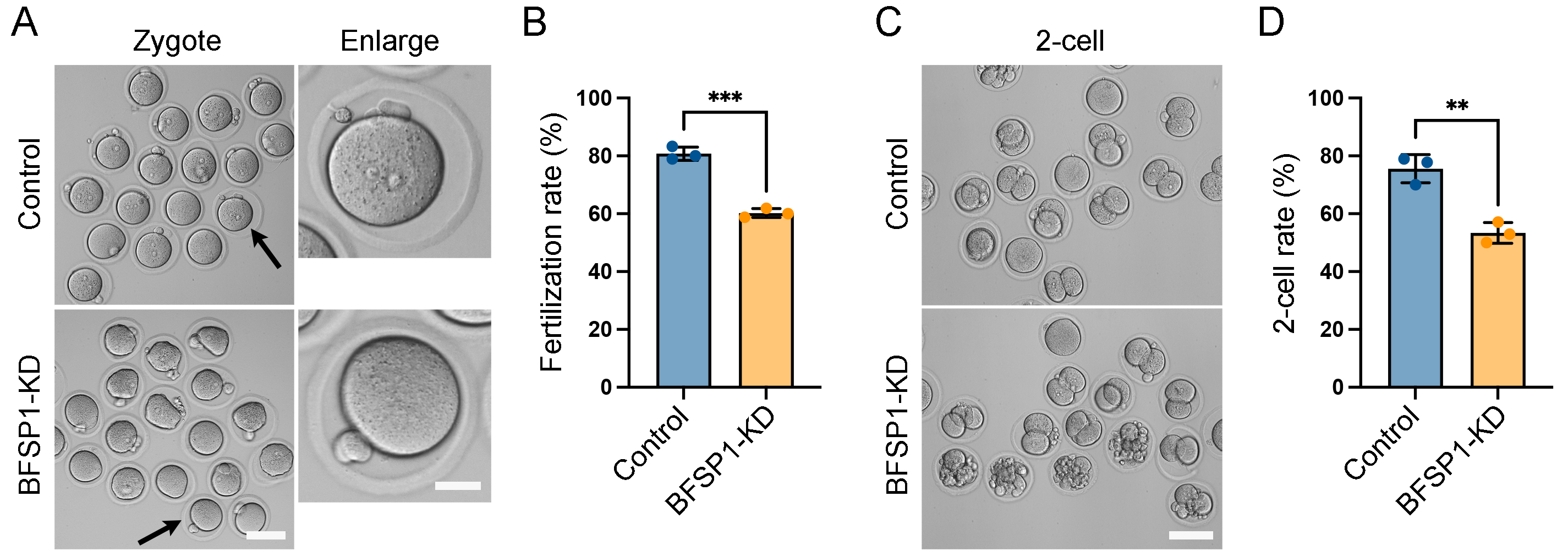


**Fig. S3. BFSP1 depletion impairs the fertilization ability of oocytes.** (A) Representative images of fertilized eggs in control and BFSP1-depleted groups. Scale bars, 80 μm, 20 μm. Oocytes at M II stage were microinjected with BFSP1 antibody (0.5μg/μl), followed by *in vitro* fertilization. (B) Fertilization rate was quantified in control (n = 57) and BFSP1-depleted (n = 58) groups. (C) Representative images of 2-cell embryos in control and BFSP1-depleted groups. Scale bar, 80 μm. (D) The proportion of 2-cell embryos was quantified in control (n = 57) and BFSP1-depleted (n = 58) groups. Data in (B) and (D) were expressed as mean ± SEM of at least three independent experiments. **P < 0.01; ***P < 0.001.


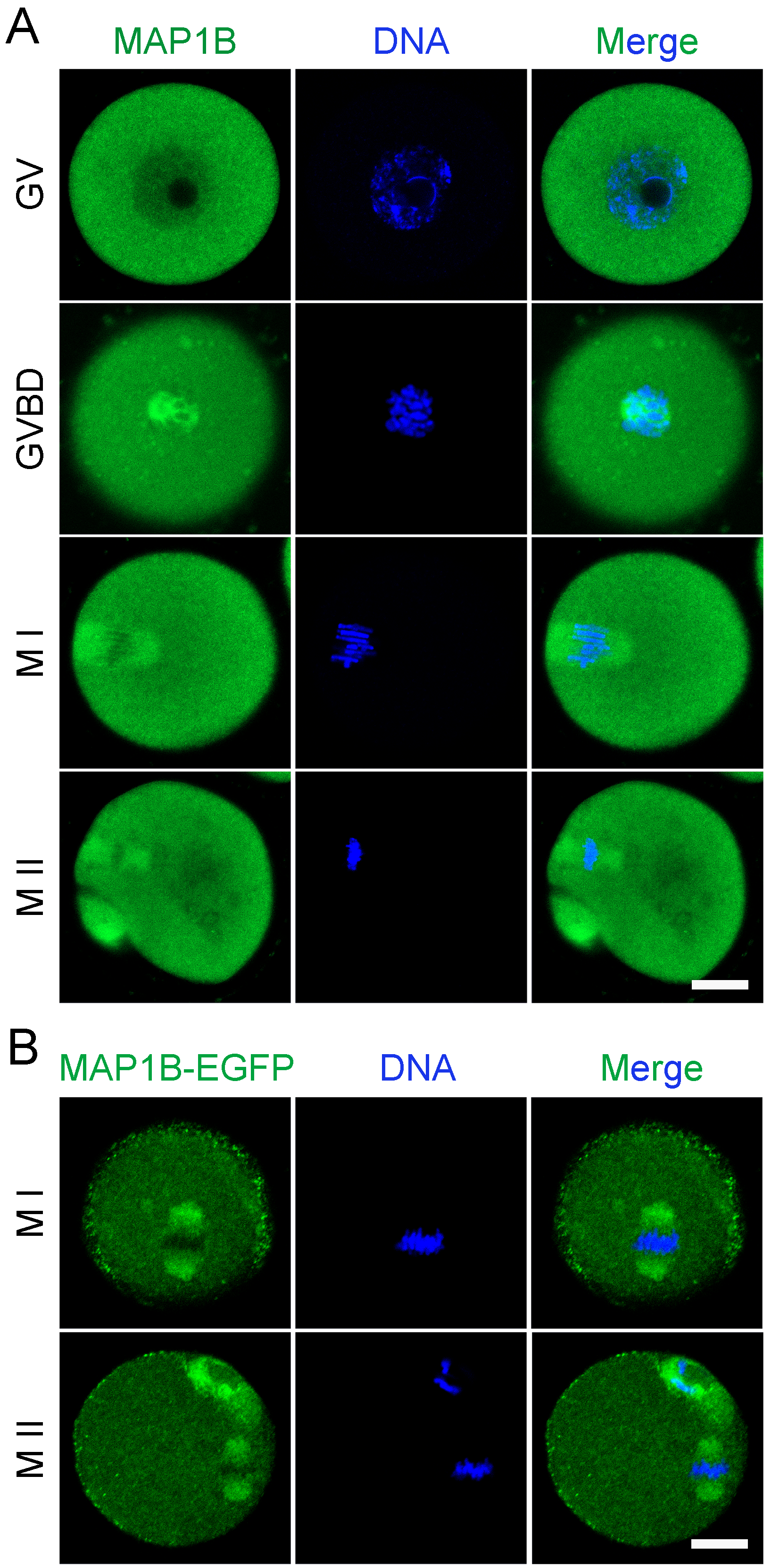


**Fig. S4. Localization of MAP1B in oocytes.** (A) Representative images of endogenous MAP1B localization during oocyte meiotic maturation. Scale bar, 20 μm. (B) Representative images of MAP1B-EGFP localization in M I and M II oocytes. Scale bar, 20 μm.


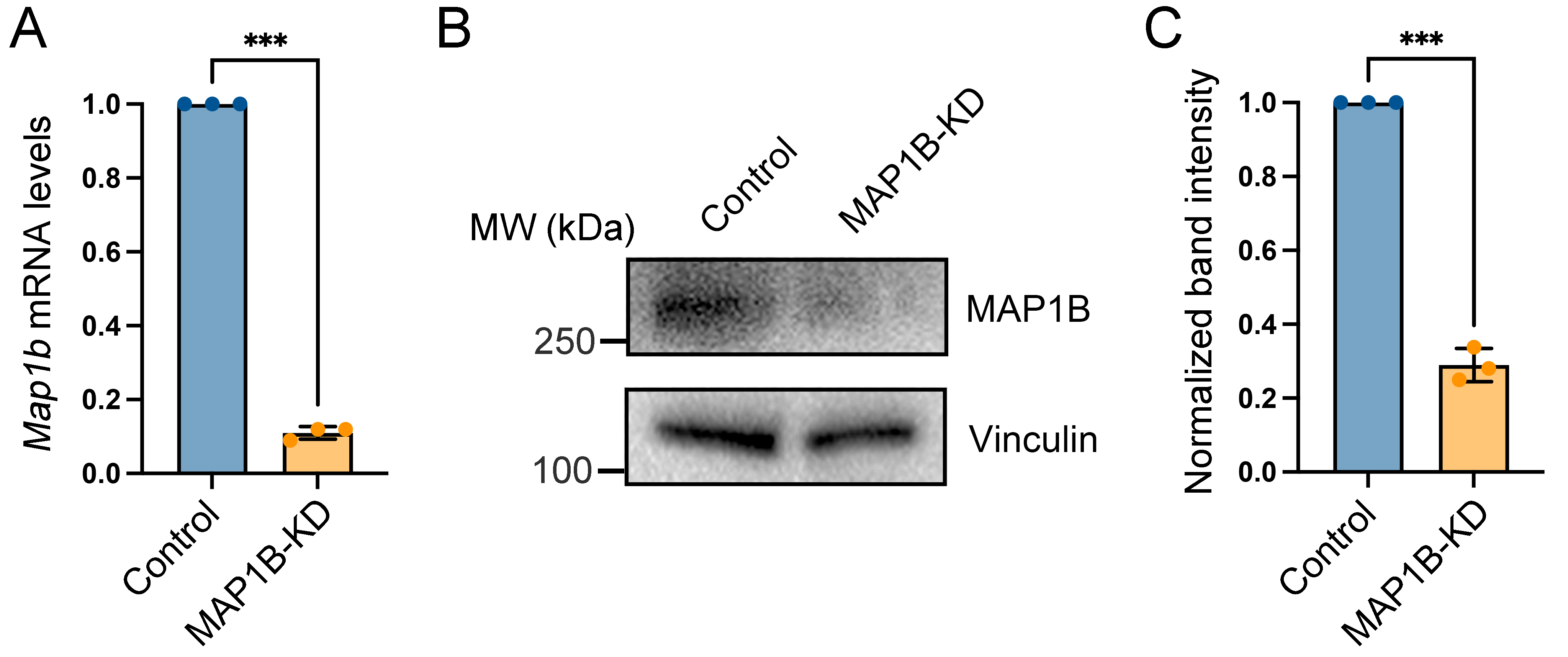


**Fig. S5. Knockdown efficiency of MAP1B in oocytes.** (A) mRNA levels of *Map1b* in control and MAP1B-KD oocytes as assessed by qRT-PCR. (B) Protein levels of MAP1B in control and MAP1B-KD oocytes as assessed by immunoblotting analysis. (C) The band intensity of MAP1B in the blots was normalized with that of Vinculin. Data in (A) and (C) were expressed as mean ± SEM of at least three independent experiments. ***P < 0.001.


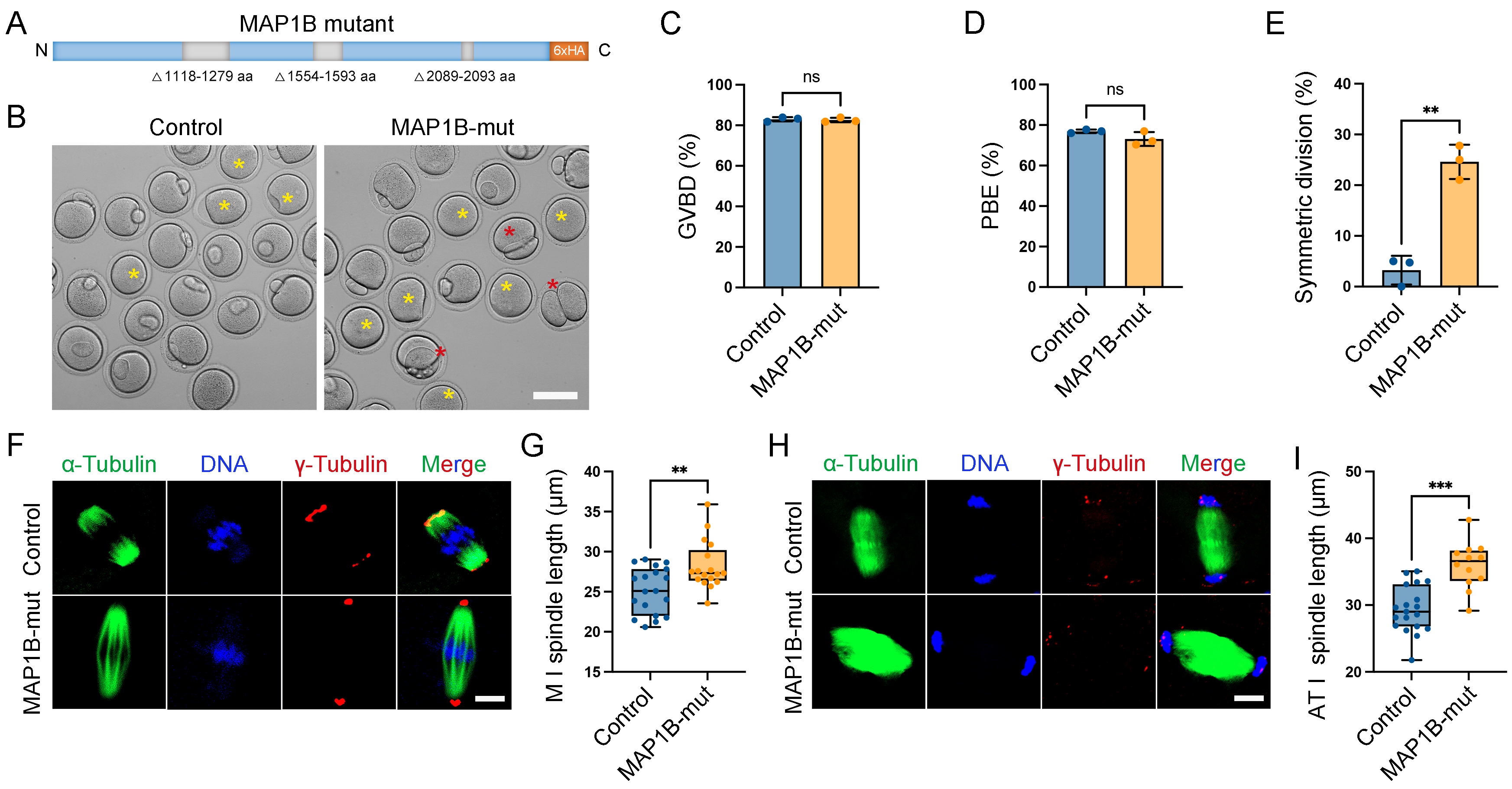


**Fig. S6. MAP1B mutant perturbs the oocyte asymmetric division and spindle length control.** (A) Schematic diagram of MAP1B construct with deletion domains required for BFSP1 binding. (B) Representative images of oocytes at M II stage in control and MAP1B-mut (expression of MAP1B deletion mutant in oocytes) groups. Yellow asterisks indicate oocytes that failed to extrude the first polar body, and red asterisks indicate oocytes with symmetric division. Scale bar, 80 μm. (C) The GVBD rate was quantified in control (n = 94) and MAP1B-mut (n = 94) oocytes. (D) The PBE rate was quantified in control (n = 94) and MAP1B-mut (n = 94) oocytes. (E) The rate of symmetric division was quantified in control (n = 94) and MAP1B-mut (n = 94) oocytes. (F) Representative images of spindle length in control and MAP1B-mut oocytes at M I stage. Oocytes were immunostained for α-tubulin and γ-tubulin. Scale bar, 15 μm. (G) Spindle length was measured between two spindle poles in control (n = 19) and MAP1B-mut (n = 17) oocytes at M I stage. (H) Representative images of spindle length in control and MAP1B-mut oocytes at AT I stage. Oocytes were immunostained for α-tubulin and γ-tubulin. Scale bar, 15 μm. (I) Spindle length was measured between two spindle poles in control (n = 19) and MAP1B-mut (n = 12) oocytes at AT I stage. Data in (C), (D), and (E) were expressed as mean ± SEM, and (G) and (I) were expressed as mean ± SD of at least three independent experiments. **P < 0.01; ***P < 0.001; ns, no significance.


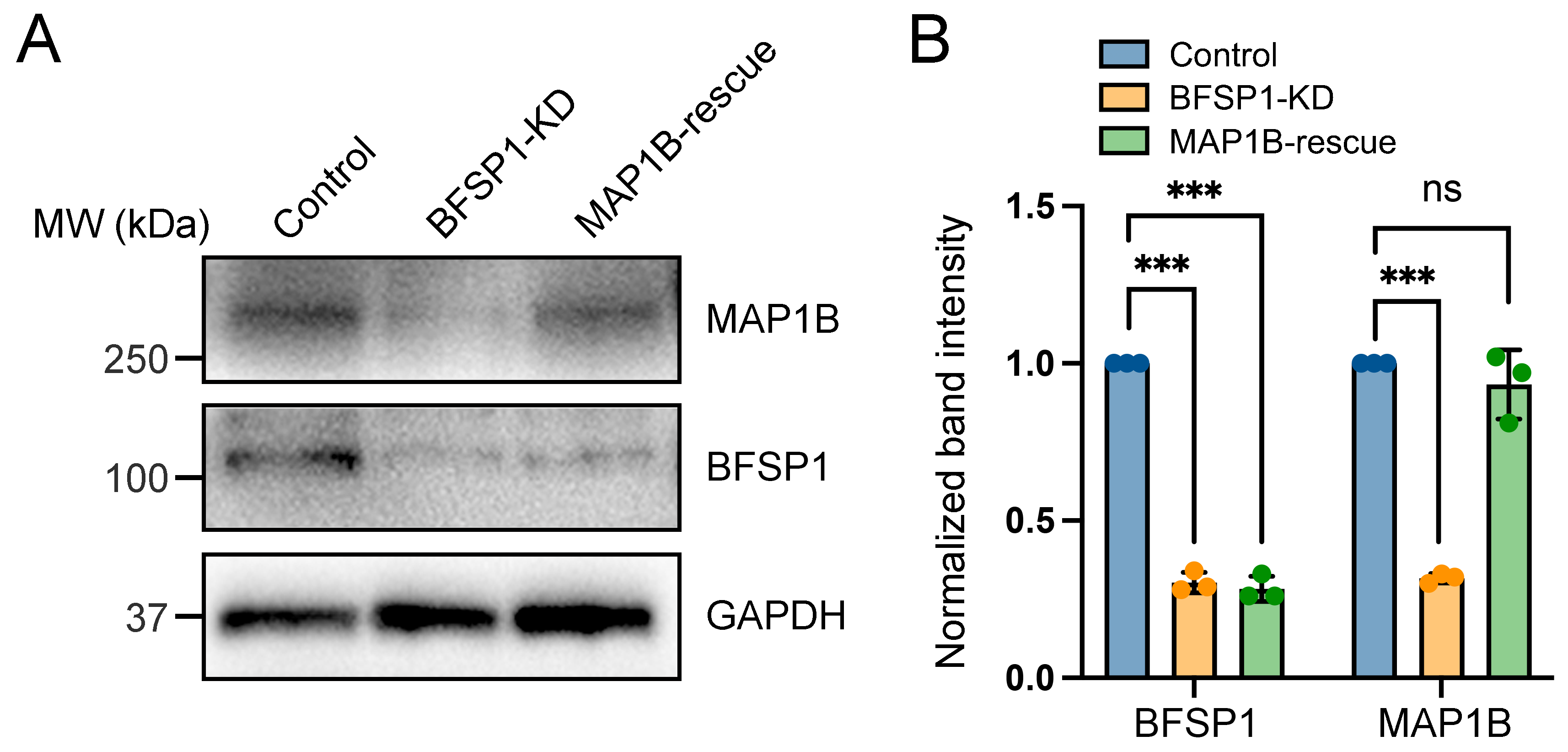


**Fig. S7. Expression of MAP1B in BFSP1-KD oocytes.** (A) Protein levels of MAP1B and BFSP1 in control, BFSP1-KD, and MAP1B-rescue oocytes as assessed by immunoblotting analysis. (B) The band intensities of BFSP1 and MAP1B in the blots were normalized with that of GAPDH. Data in (B) were expressed as mean ± SEM of at least three independent experiments. ***P < 0.001; ns, no significance.


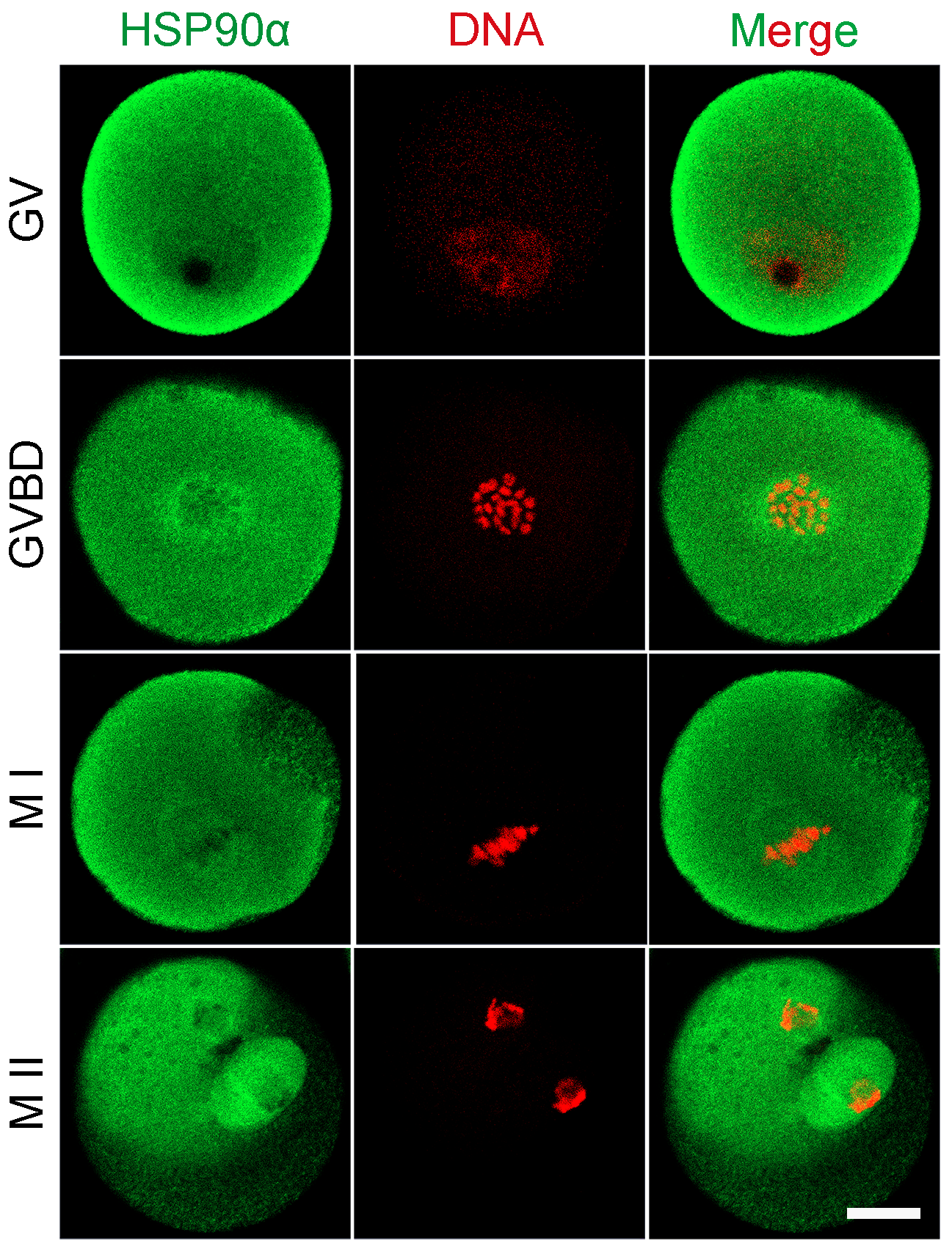


**Fig. S8. Localization of HSP90α in oocytes.** Representative images of HSP90α localization during oocyte meiotic maturation. Scale bar, 20 μm.

**
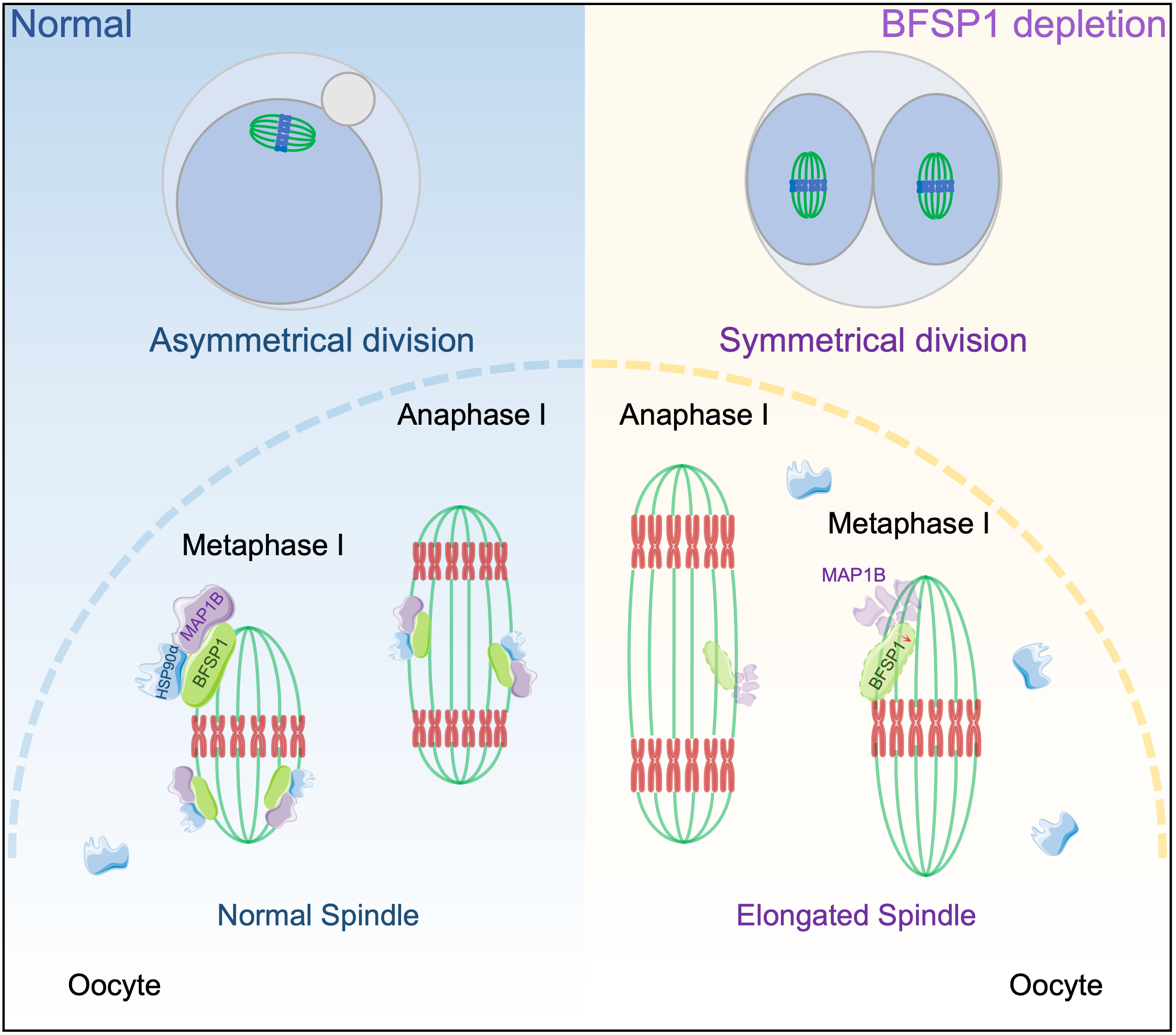
**

**Fig. S9. Working model for the control of spindle length and asymmetric division in oocytes by BFSP1.**

**Supplementary Table S1.** siRNA sequences

| **siRNA** | **Sense** | **Antisense** |
| --- | --- | --- |
| Control | UUCUCCGAACGUGUCACGUTT | ACGUGACACGUUCGGAGAATT |
| *Bfsp1* | GGAAGACUCUAUGCAUCAUTT | AUGAUGCAUAGAGUCUUCCTT |
|  | GGUACAAGAAGAAUCUUCUTT | AGAAGAUUCUUCUUGUACCTT |
|  | GGGUGGUGAUUUCCAUUGATT | UCAAUGGAAAUCACCACCCTT |
| *Map1b* | CGGGCUUCAUACCCAUAAATT | UUUAUGGGUAUGAAGCCCGTT |
|  | GCCCAAUGGUCAAGAAGUATT | UACUUCUUGACCAUUGGGCTT |
|  | GACACCCAAUGAGAUUAAATT | UUUAAUCUCAUUGGGUGUCTT |

**Supplementary Table S2. The primer sequences for q**RT-PCR

| **Gene** | **Forward Primer (5’-3’)** | **Reverse Primer (5’-3’)** |
| --- | --- | --- |
| *Gapdh* | AGGTCGGTGTGAACGGATTTG | TGTAGACCATGTAGTTGAGGTCA |
| *Bfsp1* | GGACCTGACAGCCGAGCAC | TTAGCACCAACTGACATTCACACTC |
| *Map1b* | TGCTACCTCTGGATATACTCAGTCTAC | GTCTCGTCGCTCATCACATCAC |
